# Glycosphingolipid storage in Fabry mice extends beyond globotriaosylceramide and is affected by ABCB1 depletion

**DOI:** 10.4155/fsoa-2016-0027

**Published:** 2016-10-13

**Authors:** Mustafa A Kamani, Philippe Provençal, Michel Boutin, Natalia Pacienza, Xin Fan, Anton Novak, Tonny C Huang, Beth Binnington, Bryan C Au, Christiane Auray-Blais, Clifford A Lingwood, Jeffrey A Medin

**Affiliations:** 1 University Health Network, Toronto, Ontario, M5G 1L7, Canada; 2 Department of Pediatrics, Division of Medical Genetics, Université de Sherbrooke, CHUS, Hospital Fleurimont, Sherbrooke, Quebec, J1H 5N4, Canada; 3 Division of Molecular Structure & Function, Research Institute, The Hospital for Sick Children, Toronto, Ontario, M5G 1X8, Canada; 4 Departments of Biochemistry & Laboratory Medicine & Pathobiology, University of Toronto, Toronto, Ontario, M5S 1A8, Canada; 5 Department of Medical Biophysics, Institute of Medical Sciences, University of Toronto, Toronto, Ontario, M5S 1A8, Canada

**Keywords:** ceramides, lipidomics, lysosomal storage disorders

## Abstract

**Aim:**

Fabry disease is caused by α-galactosidase A deficiency leading to accumulation of globotriaosylceramide (Gb_3_) in tissues. Clinical manifestations do not appear to correlate with total Gb_3_ levels. Studies examining tissue distribution of specific acyl chain species of Gb_3_ and upstream glycosphingolipids are lacking.

**Material & methods/Results:**

Thorough characterization of the Fabry mouse sphingolipid profile by LC-MS revealed unique Gb_3_ acyl chain storage profiles. Storage extended beyond Gb_3_; all Fabry tissues also accumulated monohexosylceramides. Depletion of ABCB1 had a complex effect on glycosphingolipid storage.

**Conclusion:**

These data provide insights into how specific sphingolipid species correlate with one another and how these correlations change in the α-galactosidase A-deficient state, potentially leading to the identification of more specific biomarkers of Fabry disease.

## Lay abstract

Fabry disease is caused by a shortage of the enzyme α-galactosidase A leading to storage of a fat called globotriaosylceramide (Gb_3_) in tissues. Disease severity does not appear to correlate directly with total Gb_3_. Importantly, Gb_3_ is comprised of many highly related but distinct species. We examined levels of Gb_3_ species and precursor molecules in Fabry mice. Gb_3_ species and storage are unique to each tissue. Furthermore, storage is not limited to Gb_3_; precursor fats are also elevated. Detailed analyses of differences in storage between the normal and α-galactosidase A-deficient state may provide a better understanding of the causes of Fabry disease.

**Figure 1.**
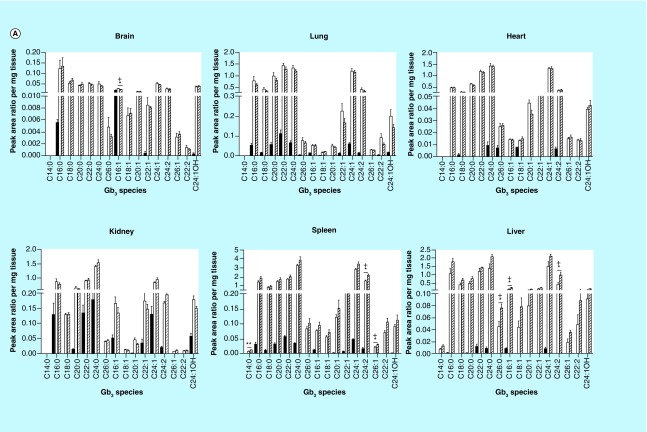

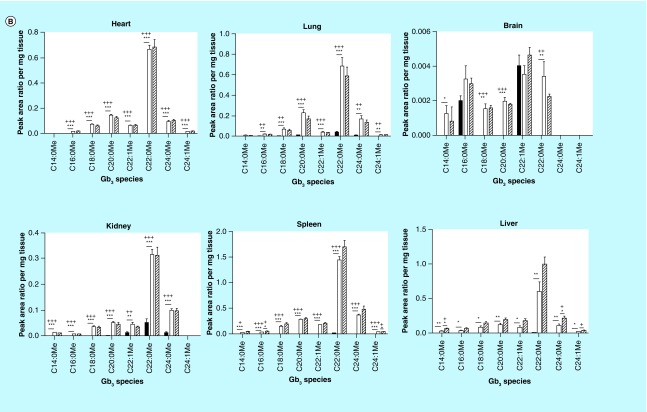
**Globotriaosylceramide acyl chain analysis.** **(A)** Glycosphingolipids were extracted from frozen tissues, separated by UPLC, and Gb_3_ acyl chain species were analyzed by MS. All analyzed species varying in chain length, saturation and hydroxylation were significantly elevated in Fabry mice relative to wild-type (WT). **(B)** Most *N*-methylated Gb_3_ species analyzed were undetectable in WT tissues, with elevated levels observed in most Fabry tissues. Dark, open and striped bars correspond to WT, Fabry and MF, respectively. *,+ p < 0.05; **,++ p < 0.01; ***,+++ p < 0.001 based on the student's *t*-test (*) or one-way ANOVA followed by the Bonferroni post-test (+); n = 4. ANOVA: Analysis of variance; Gb_3_: Globotriaosylceramide; MF: MDR1a/b/Fabry mouse; MS: Mass spectrometry.

**Figure 2.**
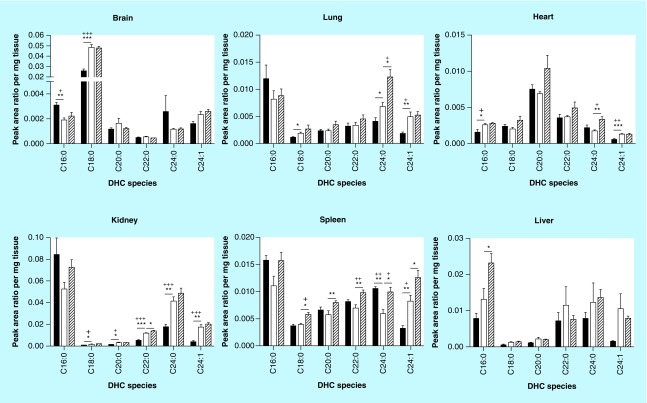
**Dihexosylceramide acyl chain species analysis.** Glycosphingolipids were extracted from frozen tissues, separated by HPLC, and DHC acyl chain species were analyzed by MS. DHC expression profiles vary based on tissue. DHC species are either elevated or unchanged in MF relative to Fabry mouse tissues. Dark, open and striped bars correspond to wild-type, Fabry and MF, respectively. *,+ p < 0.05; **,++ p < 0.01; ***,+++ p < 0.001 based on the student's *t*-test (*) or one-way ANOVA followed by the Bonferroni post-test (+); n = 4. ANOVA: Analysis of variance; DHC: Dihexosylceramide; MF: MDR1a/b/Fabry mouse.

**Figure 3.**
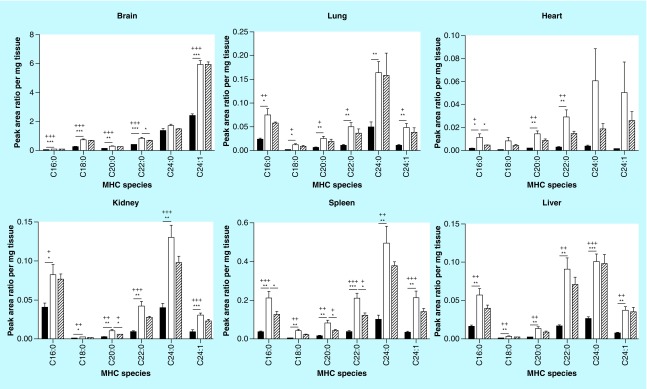
**Monohexosylceramide acyl chain species analysis.** Glycosphingolipids were extracted from frozen tissues, separated by HPLC, and MHC acyl chain species were analyzed by MS. MHC expression profiles show tissue-specific patterns. All species in all tissues are increased in Fabry mice relative to wild-type. MHC levels are either reduced or unchanged in MF relative to Fabry mouse tissues. Dark, open and striped bars correspond to wild-type, Fabry and MF, respectively. *,+ p < 0.05; **,++ p < 0.01; ***,+++ p < 0.001 based on the student's *t*-test (*) or one-way ANOVA followed by the Bonferroni post-test (+); n = 4. ANOVA: Analysis of variance; MF: MDR1a/b/Fabry mouse; MHC: Monohexosylceramide; MS: Mass spectrometry.

**Figure 4.**
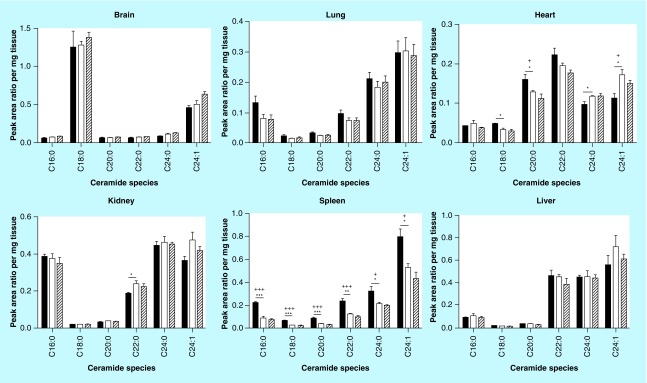
**Ceramide acyl chain species analysis.** Glycosphingolipids were extracted from frozen tissues and separated by HPLC. Ceramide acyl chain species were analyzed by MS. A reduction in ceramides was observed in the Fabry spleen relative to wild-type. No significant changes were seen between MF and Fabry tissue ceramides. Dark, open and striped bars correspond to wild-type, Fabry and MF, respectively. *,+ p < 0.05; **,++ p < 0.01; ***,+++ p < 0.001 based on the student's *t*-test (*) or one-way ANOVA followed by the Bonferroni post-test (+); n = 4. ANOVA: Analysis of Variance; MF: MDR1a/b/Fabry mouse.

**Figure 5.**
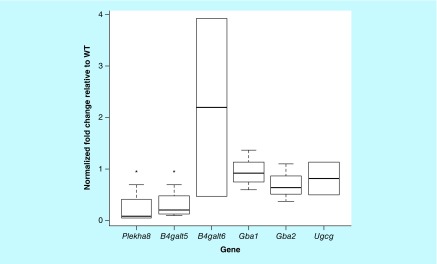
**mRNA levels of genes involved in glycosphingolipid metabolism.** Transcript levels in the liver of WT and Fabry mice were assessed by real-time quantitative reverse-transcription PCR. Data shown are normalized to endogenous housekeeping control genes (actin and/or GAPDH) and expressed as fold-change relative to WT. A significant decrease in *Plekha8* and *B4Galt5* was observed in the Fabry liver. WT: Wild-type.

**Figure 6.**
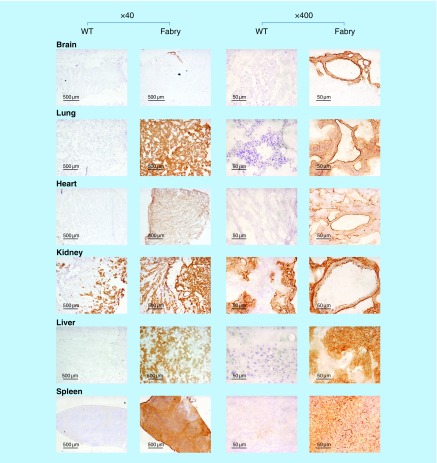
**Tissue-wide accumulation of globotriaosylceramide in Fabry mice.** Tissue globotriaosylceramide levels were evaluated by histochemical staining using verotoxin. Sections were treated with MCD to deplete cholesterol. Staining was markedly higher in Fabry tissues and was observed throughout the tissue sections. MCD: Methyl-β-cyclodextrin; WT: Wild-type.

Fabry disease (OMM 301500) is an X-linked lysosomal storage disorder caused by a deficiency in α-galactosidase A (α-gal A, EC 3.2.1.22) activity. This deficiency leads to a progressive deposition of glycosphingolipids (GSLs) with terminal α-galactose linkages, predominantly globotriaosylceramide (Gb_3_), throughout the body [1]. Such accumulations lead to life-threatening complications with patients typically suffering from chronic pain, skin lesions, renal insufficiency, cardiomyopathy and early death [1–3]. Interestingly, Fabry disease clinical manifestations and severity do not necessarily correlate with Gb_3_ levels [4,5], creating a gap in our understanding of how the molecular defects lead to the observed pathology. Importantly, Gb_3_ does not simply refer to a singular molecular species; rather, several species exist that vary in acyl chain composition.

In recent years, it has increasingly become apparent that the seemingly subtle differences within the acyl chain of sphingolipids belie unique functional roles [6–9]. Indeed, reports showing a lack of correlation of Fabry disease pathology with Gb_3_ accumulation have based their conclusions on total Gb_3_ levels and not specific acyl chain species. A notable exception to this is lyso-Gb_3_, which lacks an acyl chain and therefore does not have such species. Even for this species, however, Fabry disease pathology does not seem to correlate directly with lyso-Gb_3_ accumulation, as young patients and newborns, who do not display noticeable symptoms, show markedly elevated lyso-Gb_3_ levels [5]. Unlike what happens in humans, Fabry mice deficient in α-gal A activity display little, if any, disease phenotype, a rather surprising finding given that there is still substantial substrate accumulation in the mouse tissues [10–12]. Despite the lack of a prominent phenotype, however, this mouse model continues to be used in studies exploring novel therapeutic strategies [13–17]

Although enzyme replacement therapy is currently available for Fabry disease, the high cost of this treatment (in excess of US$250,000/patient per year) [18,19], its adverse effect of stimulating an immune response against the infused enzyme [20] and its variable clinical response [21] have prompted exploration of alternative therapeutic approaches, such as molecular chaperone therapy [22,23], gene therapy [24] and substrate reduction therapy (SRT) [25]. SRT has typically involved inhibition of glucosylceramide (GlcCer) synthase, the first enzyme in the glucose-based GSL biosynthesis pathway [25]. However, alternative approaches have also been proposed, such as inhibition of the multidrug resistance efflux pump 1 or P-glycoprotein (MDR1, P-gp, ABCB1) [26].

ABCB1 is the archetypal member of the ATP-binding cassette transporter superfamily of proteins. It mediates the cellular efflux of a broad range of hydrophobic substrates [27]. Although well known for its role in conferring chemoresistance to tumor cells, ABCB1 also plays an important role in normal physiology by protecting tissues from toxic xenobiotics and endogenous metabolites [28]. The human and murine MDR1 genes, *MDR1* and *Mdr1a/b*, respectively, are highly expressed in the intestinal epithelium, adrenal gland, brain and testis [29,30]. ABCB1 is also a key component of the blood–brain and blood–testis barriers [31]. In addition to its localization to the cell surface, ABCB1 is also found in the Golgi and lysosomal membranes [32]. Our group and others have shown that an ABCB1 flipping mechanism can facilitate the translocation of GlcCer from its site of synthesis on the cytoplasmic leaflet of the Golgi apparatus to the luminal leaflet for access to downstream glycosyltransferases, mediating a key step in GSL biosynthesis [33–40]. We have demonstrated that ABCB1 inhibition depletes cells of Gb_3_ by preventing its *de novo* synthesis, and that Fabry mice treated by enzyme replacement therapy followed by administration of cyclosporine A, an ABCB1 inhibitor, failed to accumulate Gb_3_ in the liver, suggesting that inhibition of ABCB1 may have therapeutic consequences for Fabry disease patients [26].

In this study, we examined the Fabry mouse tissue content of GSL species varying in acyl chain composition in an effort to discern whether there is a differential accumulation profile of Gb_3_ species and to understand how α-gal A deficiency affects other GSLs in the Gb_3_ biosynthetic pathway. This will help us understand the relationships between specific sphingolipid species in the normal and α-gal A-deficient state, and may thereby lead to the identification of more specific biomarkers of Fabry disease pathology – and, therefore, therapy. Concurrently, we generated a novel knockout MDR1a/b/Fabry (MF) mouse and characterized lipid accumulation in tissues from that model. This triple knockout (*Mdr1a/Mdr1b/Gla*) model allowed us to directly evaluate the therapeutic potential of targeting this protein to reduce Gb_3_ levels.

## Materials & methods

### MF mouse generation

α-Gal A-deficient Fabry mice (C57BL/6; 129/SvJ background) [10] were bred at the Animal Resource Centre, University Health Network (UHN). MDR1a/b mice (FVB background) were purchased from Taconic (NY, USA) and bred in a colony maintained at UHN. Animal experimentation protocols were approved by the UHN Animal Care Committee. The parental generation (F0) involved in the genesis of the MF mice consisted of Fabry females (AABBxx) crossed with MDR1a/b male (aabbXY) mice. In order to generate the four different genotypes analyzed in the present study (wild-type [WT], Fabry, MF, and MDR) (Supplementary Figure 1), the F_1_-triple heterozygous mice were mated (AaBbXx by AaBbxY). Each of the genotypes was found in the expected ratio. Mice were healthy, had similar growth rates and no untoward gross physiological differences were seen. At the age of 23–27 weeks, male mice were euthanized and their organs of interest (spleen, liver, kidney, brain, lung and heart) were isolated and immediately frozen until processing.

### Genotyping

Mouse genotypes were identified by analyzing DNA from tails or notched ear pieces. *Mdr1a* genotype identity was determined using Taconic's recommendations: a single PCR reaction using three primers was sufficient to identify the two possible *Mdr1a* alleles (WT 269 bp and mutant 461 bp). The murine *Mdr1a* and *b* genes are linked and, therefore, transmit ligated. Correspondingly, the allelic states of both these genes are identical and genotyping of the *Mdr1b* gene was not always performed. WT *Mdr1b* (540 bp) was assessed following recommendations by Taconic. New sets of primers were designed to determine the mutated *Mdr1b* (HS5-forward 5′TGTCAAGACCGACCTG TCCG3′ and NeoB-Reverse 5′ACGCGTCGCGACGCGTCTAG3′), yielding a product of 1127 bp, and WT and mutated *α-gal A* alleles (GLA-F1 5′TCCTGGTTGGTTTCCTATTGTGG-3′, GLA-R1 5′TCTGACTTCTCAACAGGCACCATAG and Neo-R1 5′TGTGCCCAGTCATAGCCGAA-3′) with product sizes of 327 and 714 bp, respectively.

### α-Gal A activity assay

Specific α-gal A activity was determined by fluorometric assay as previously described [41]. Briefly, organ protein extracts were incubated with 4-methylumbelliferyl-α-d-galactopyranoside (5 mM) (RPI Corp., IL, USA) in the presence of the α-*N*-acetyl-galactosaminidase inhibitor, *N*-acetyl-d-galactosamine (100 mM) (Sigma, ON, Canada) [42,43]. The product of the enzymatic reaction was quantified by comparison with known concentrations of 4-methylumbelliferone. Each measurement was assessed in triplicate, normalized to total protein concentration (DC^TM^ (Detergent-Compatible) Protein Assay, Bio-Rad Laboratories, ON, Canada), and expressed as mean specific activity ± SD.

### Mass spectrometry

For monohexosylceramide (MHC) and dihexosylceramide (DHC) MS analyses, tissue samples were homogenized in water (weight by volume [1:8]) with Omni Bead Ruptor 24 (Omni International, Inc., GA, USA). The glycosylceramides were extracted with 500 μl of methanol from 50 μl of each of the tissue homogenates. Galactosylceramide (d18:1/C8:0) (58.8 ng) (Avanti Polar Lipids, AL, USA) and deuterated DHC (d18:1/C16:0)D3 (470 ng) (Matreya LLC, PA, USA) were used as internal standards for corresponding MHCs and DHCs, respectively, and added to the samples before extraction. Sample analyses were performed with a Shimadzu 20AD HPLC system and a Leap PAL autosampler coupled to a triple quadrupole mass spectrometer (API 4000: Applied Biosystems, ON, Canada) operated in MRM mode. The positive-ion ESI mode was used for detection of glycosylceramides. These study samples were injected in duplicate for data averaging. Data processing was conducted with Analyst 1.5.1 (Applied Biosystems). The relative quantification of lipids is provided, and the data are reported as the peak area ratios of the analytes to the corresponding internal standards. Bicinchoninic acid (BCA) assays were performed on all the tissue samples for protein determination.

Ceramides were extracted from 50 µl of each of previously homogenized tissue with 250 µl of isopropanol. Deuterated ceramide (d18:1/C22:0)d4 (100 ng; synthesized internally in the Metabolomics Facility at Washington University) was used as the internal standards for ceramide analyses and added to the samples before extraction. Quality control samples were prepared from pooling some tissue extracts. The sample analysis was performed with a Shimadzu 20AD HPLC system and a Leap PAL autosampler coupled to a triple quadrupole mass spectrometer (API 4000: Applied Biosystems) operated in MRM mode. The positive-ion ESI mode was used for detection of ceramides. These tissue extract samples were injected in duplicate for data averaging. Data processing was conducted with Analyst 1.5.1 (Applied Biosystems).

For Gb_3_ analyses by MS, tissues from each organ were homogenized in methanol using an Omni Bead Ruptor 12 (VWR, ON, Canada) to obtain a concentration of 100 mg of tissue per ml of methanol. Tissues were extracted using a method previously published by the Sherbrooke group for the analysis of Gb_3_ in plasma [44]. Briefly, 30 µl of deuterated *N*-octadecanoyl-globotriaosylceramide (Gb_3_-d18:1/C18:0)D3 (1 µg/µl) internal standard (Matreya LLC, PA, USA), 3 ml of methanol and 1.5 ml of CHCl_3_ were added to 200 µl of sample homogenate. Incubation was performed for 15 h at 48°C. Thereafter, 450 μl of 1 M KOH (methanolic) was added for the hydrolysis of molecules which might interfere with the liquid–liquid extraction, and the mixture was incubated for 2 h at 37°C. Solutions were neutralized with 18 μl of glacial acetic acid. For liquid–liquid extraction, 2 ml of CHCl_3_ and 4 ml of water were added; the tubes were vortexed, sonicated and centrifuged for 5 min at 5000 rpm. The lower organic phase was collected. A second extraction of the aqueous phase was performed by adding 2 ml of CHCl_3_. The two organic phases were combined and dried under a nitrogen stream. Samples were resuspended with 100 μl of CH_3_OH/5 mM ammonium formate/0.1% formic acid. Samples were separated by ultra-HPLC (UPLC; Acquity, Waters Corp., MA, USA) and analyzed by TOF MS using a ESI-QTof-MS (Synapt G1, Waters) according to methods previously published [44,45].

For MS analyses of gangliosides, tissue samples were homogenized in water (weight by volume [1:8]) with Omni Bead Ruptor 24 (Omni International, Inc.). Gangliosides were extracted with 500 µl of methanol from 100 µl of the tissue homogenate. N-CD_3_-Stearoyl-GM3 (400 ng) and N-D_3_-Stearoyl-GM1 (400 ng; Matreya, LLC) were used as internal standards for corresponding ganglioside classes and added to the samples before extraction. Sample analysis was performed with a Shimadzu 20AD HPLC system, a Leap PAL autosampler coupled to a triple quadrupole mass spectrometer (API 4000) operated in MRM mode. The negative-ion ESI mode was used for detection of gangliosides. The study samples were injected in duplicate for data averaging. Data processing was conducted with Analyst 1.5.1 (Applied Biosystems). The relative quantification of lipids is provided, and the data are reported as the peak area ratios of the analytes to the corresponding internal standards. BCA assays were performed on all the tissue samples for protein determination.

Quality control data are provided in Supplementary Table 1, showing the reproducibility of analyses, presented as coefficient of variation (CV%). Coefficient of variation less than 15% is considered acceptable. Since most of the measured analytes are not commercially available, data were reported as the ratio of the relative concentration of the analyte to the internal standard. Thus, absolute quantifications could not be performed, and instead of LOD and LOQ, LLOQ is shown in Supplementary Table 2. Data obtained are higher than LLOQ and are, therefore, considered valid. For Gb_3_, only peaks showing a signal-to-noise ratio greater than 10 were evaluated (the standard criteria for LOQ determination). Sample chromatograms are shown in Supplementary Figure 2.

MS analyses for MHCs, DHCs and gangliosides were performed in the Metabolomics Facility at Washington University headed by Dr Daniel Ory (P30 DK020570).

### Quantitative real-time-PCR

A small piece of frozen tissue was subjected to TRIzol^®^ (Life Technologies, CA, USA) for isolation of RNA, as per the manufacturer's protocol. 2 µg of RNA was treated with DNase I (ThermoFisher Scientific, MA, USA) and reverse transcribed to CDNA using RevertAid H Minus Reverse Transcriptase and oligo-dT primers (ThermoFisher Scientific). Real-time quantitative reverse-transcription PCR (qRT-PCR; qPCR) was performed using the ABI 7900HT Fast RT-PCR system (Applied Biosystems), Sybr Green (ThermoFisher Scientific) and primers designed to specifically hybridize to the corresponding genes of interest. Relative gene expression was calculated for WT and Fabry samples using expression standard curves and normalization to endogenous controls (β-actin and/or glyceraldehyde 3-phosphate dehydrogenase [GAPDH]). Then, expression fold-changes of Fabry samples were calculated over the WT samples.

### Gb_3_ staining of tissue sections

Tissue Gb_3_ levels were also evaluated by verotoxin 1 (VT1) staining and immunohistochemistry. VT1 staining was performed as described [26]. Briefly, frozen tissue cryosections were air-dried overnight, blocked with endogenous peroxidase blocker (Universal Block; KPL, Inc., MD, USA) and then stained with VT1-B (1 μg/ml) as described [46]. For some sections, cholesterol was extracted by treatment with 10 mM methyl-β-cyclodextrin [47] for 30 min at 37°C prior to staining. After rinsing, sections were incubated with rabbit anti-VT1B 6869 [48], washed and then incubated with goat anti-rabbit IgG conjugated with horseradish peroxidase (HRP) (Bio-Rad, CA, USA). After washing, sections were developed using the DAB (3,3′-diaminobenzidine) substrate (Vector Labs, Inc., CA, USA). For immunodetection of Gb_3_, an additional avidin/biotin blocking step was added: tissue sections were incubated for 30 min with a rat monoclonal anti-Gb_3_ (clone 38.13), washed and then incubated with biotin anti-rat IgM (Jackson Immunoresearch, PA, USA). Staining was developed using ATP-binding cassette Elite DAB stain (Vector Labs). Specificity of Gb_3_ detection by VT1 or 38.13 was verified by preparing control sections in which VT1 was omitted, or isotype control rat IgM (eBioscience, CA, USA) was substituted, respectively. Following DAB staining, sections were counterstained with hematoxylin and then mounted with Permount (Fisher Scientific, ON, USA).

### Statistical analyses

Sphingolipid data have been expressed as mean ± standard error of the mean (SEM) with 3–5 mice per group. Differences between groups were assessed by one-way analysis of variance (ANOVA), followed by a Bonferroni post-test and two-tailed homoscedastic *t*-tests. Values of p < 0.05 were considered to be statistically significant. For qPCR analysis, statistical significance was evaluated using a one-sample two-tailed *t*-test with an expected mean of one. Correlation analyses were performed using GraphPad Prism. Raw LC-MS data were input and a matrix of correlation coefficients and corresponding p-values (two-tailed, 95% CI) was constructed. Statistically significant differences between WT and Fabry correlations were assessed by applying Fisher's *Z*-transformation to correlation coefficients [49].

## Results

Target mice were generated by crossbreeding Fabry and *Mdr1a/b* knockout mice. After ascertaining the mouse genotypes, we confirmed the Fabry phenotype by assessing tissue α-gal A activity. As expected, WT and MDR mice showed comparable levels of α-gal A activity that was much higher than that in tissues from both Fabry and MF mice (Supplementary Figure 1).

### LC-MS analysis of GSLs

We performed in-depth LC-MS analyses of Gb_3_ in six tissues from WT and Fabry mice in an effort to examine the distribution of specific Gb_3_ species. We assessed levels of 24 Gb_3_ species varying in acyl chain length, saturation, hydroxylation and in *N*-methylation of the sphingosine backbone. Each Fabry tissue had substantially higher levels of almost all Gb_3_ species relative to the corresponding WT tissues (Figure 1A), with the exception of a few rare methylated Gb_3_ species (Figure 1B). Importantly, we observed differential expression of Gb_3_ species between different tissues of mice with the same genotype. WT brain and heart showed very limited Gb_3_ profiles, with only a few detectable species, while the lung and spleen showed the most diverse profiles among WT tissues. This was in stark contrast with the α-gal A-deficient state, wherein Fabry tissues expressed detectable levels of many species that were undetectable in WT tissues (Figure 1). Within a given tissue, the fold-change of each Gb_3_ species in Fabry mice relative to WT was also unique. In addition, there was a differential accumulation of given Gb_3_ acyl chain species in Fabry mice across the tissues examined. For each Gb_3_ species, a large range of fold-changes were observed across the six tissues, from undetectable changes of a species in a particular tissue to several hundred-fold elevation of the same species in a different tissue.

In addition to the changes in particular acyl chain species, large changes were observed in the percent compositions of many Gb_3_ species (as a fraction of total analyzed Gb_3_) when comparing tissues of WT and Fabry mice (Table 1). The percent abundance of many of the more prevalent Gb_3_ species in WT tissues was markedly reduced in Fabry mice. These reductions were, in part, offset by elevations in species not detected in WT samples. For example, in the brain, a 57% reduction was seen in the C16:1 Gb_3_ proportion in Fabry mice. In the heart and liver, this species was only a minute proportion of total Gb_3_ (0.2 and 1.5%, respectively), while it represented almost 20–25% of total Gb_3_ in the corresponding WT tissues. Concurrently, we examined Gb_3_ content in MF tissues. Most Gb_3_ species were unaltered relative to Fabry mice; however, some significant increases were observed in the liver and spleen (Figure 1 & Table 2). No general trend was observed in comparing specific Gb_3_ species levels between WT and MDR tissues (data not shown).

### MHCs & DHCs

Next, we sought to examine the effect of α-gal A deficiency on upstream GSLs in the biosynthetic pathway, thereby providing a detailed characterization of these GSLs in an effort to move toward characterization of the glycosphingolipidome of the Fabry mouse. We sought to evaluate levels of the neutral GSL precursors of Gb_3_: GlcCer and lactosylceramide (LacCer). Since GlcCer and galactosylceramide are of identical mass, the data reported herein reflect both species that are collectively referred to as MHCs. Similarly, LacCer and galabiosylceramide have an identical mass; the data reported represent both species collectively referred to as DHCs. A mix of elevations and reductions in Fabry mouse DHCs relative to WT were seen in the analyzed species (Figure 2 & Table 3): significant elevations were seen in Fabry brain C18:0; heart C16:0 and C24:1; kidney C18:0, C20:0, C22:0, C24:0 and C24:1; lung C18:0, C24:0 and C24:1; and spleen C24:1. A significant reduction was detected in brain C16:0 and spleen C24:0. Depletion of ABCB1 had the greatest effect on spleen, with all species being elevated in MF mice relative to Fabry mice. Other tissues showed no more than a single species significantly increased in MF mice. In terms of the percent composition of each DHC species, several notable differences were observed (Table 4). An elevation of C18:0 DHC in the Fabry brain was accompanied by reductions in the percentage of C16:0 and C24:0; an approximately 30% reduction in kidney C16:0 was offset by elevations in C22:0, C24:0 and C24:1 DHC; increases in liver C16:0 and C24:1 DHCs were offset by reductions in C22:0 and C24:0; a 25% reduction in lung C16:0 was seen along with increases in C24:0 and C24:1 DHCs; reductions in C16:0 and C24:0 DHCs were accompanied by an increase in C24:1.

With regard to MHCs, all species evaluated were significantly elevated in the Fabry mouse (Figure 3). Unlike the fold-changes seen within a tissue for the various Gb_3_ species, the MHC species’ fold-changes were generally similar (Table 3). In contrast, a greater variation in fold-change accumulation of a given MHC species was observed across tissues. In MF mice, a few MHC species were altered: brain C22:0, heart C16:0, kidney C20:0 and spleen C16:0, C20:0 and C22:0 were all significantly reduced, with all others being unchanged. The composition of each MHC as a percentage of total MHCs was, for the most part, retained between WT and Fabry tissues (Table 5), a finding that is in-line with the similar fold-changes observed for all species within a tissue (Table 3). Notable exceptions included a 12% reduction in C24:0 in the Fabry brain relative to WT, which was mostly offset by an elevation of C24:1 MHC; a 17% increase in heart C24:1 that was partially offset by reductions in C24:0, C22:0 and C16:0 MHCs; a 12% reduction in kidney C16:0 was partially offset by modest increases in the percent compositions of multiple MHC species.

### Ceramides

We extended the evaluation of GSLs back to the precursor of all GSLs- ceramide- and examined levels of ceramide species by LC-MS. All Fabry spleen ceramides were reduced relative to WT, while variable increases and reductions were observed in Fabry heart ceramides compared with WT (Figure 4 & Table 3). Other tissues exhibited similar levels of all ceramides assessed. No significant differences were observed in MF tissue ceramides relative to Fabry tissues.

### Sphingolipid metabolic correlations

We delved deeper into the analysis of LC-MS in an attempt to identify any patterns between species of the same lipid type within and across tissues, as well as between lipid types for a given acyl chain within tissues. To address these points, a series of correlation matrices were generated (Supplementary Tables 3, 4, 5 & 6). WT mice exhibited limited Gb_3_ correlations within a given tissue, but several positive and negative correlations when comparing species across tissues. By contrast, Fabry mice showed extensive Gb_3_ species correlations both within and across tissues. WT and Fabry mouse tissues showed some strong correlations between DHC species. WT tissues showed a greater number of correlations between MHC species and between ceramide species than did Fabry tissues.

In order to compare the differences between the correlations identified for WT mice sphingolipid species and the corresponding Fabry mouse species, Fisher's method was used to transform the correlations to a linear scale. Several significant differences were identified between correlation coefficients of the two groups of mice (Supplementary Tables 7, 8, 9 & 10). Within a given tissue, the greatest number of significant differences between WT and Fabry Gb_3_ correlations was seen in the liver, while limited significant differences were observed when comparing correlations across tissues. Fewer significant differences between correlations were observed for DHCs. For MHCs, essentially no significant differences existed between WT and Fabry correlations within a given tissue; however, a large number of significant differences were observed for heart MHC correlations with kidney and lung MHCs. The heart was also the tissue with the greatest number of significant differences for ceramide correlations.

Having acquired LC-MS data for sequential analytes in a metabolic pathway (i.e., ceramide → MHC → DHC → Gb_3_), we evaluated the data for correlations of particular acyl chains across sphingolipid type within a given tissue. Our analysis revealed that sphingolipid species in Fabry tissues had a greater number of strong correlations to different lipid types with the same acyl chain, compared with that in WT (Supplementary Tables 11, 12, 13, 14, 15 & 16). A few of these correlations were significantly different between WT and Fabry mice (Supplementary Tables 17, 18, 19, 20, 21 & 22).

### GSL metabolism enzyme transcript levels

We performed qRT-PCR on mRNA extracted from the liver of Fabry and WT mice in order to examine whether the observed differences in sphingolipid levels can be explained by alterations in the transcript levels of genes involved in sphingolipid metabolism, namely GlcCer synthase (*Ugcg*), the lysosomal and nonlysosomal glucosylceramidases *Gba1* and *Gba2*, respectively, the two LacCer synthases, *B4Galt5* and *B4Galt6*, and *Plekha8*, which encodes the protein FAPP2, shown to be involved in GlcCer access to the Golgi lumen [50,51]. Significant reductions in *B4Galt5* and *Plekha8*, and a modest reduction in *Gba2*, were observed, while *Gba1* transcript levels were unchanged (Figure 5).

### Fabry mice show tissue-wide elevations in Gb_3_

The MS analyses of GSL acyl chain species (above) were performed on whole tissue extracts. To begin to probe into the regional distribution of Gb_3_ in the tissues of interest, histochemistry was performed on tissues from the target groups of mice. Staining was performed using VT1 (Figure 6) and, in some cases, a monoclonal antibody against Gb_3_ (data not shown). Cell and tissue GSL staining can be greatly influenced by membrane cholesterol, which has been shown to confer a membrane parallel conformation of the GSL glycans that are not easily bound by their ligands [52]. Cholesterol depletion renders the glycan more accessible to ligands. We extracted cholesterol by treating sections with methyl-β-cyclodextrin (MCD). Staining of Gb_3_ was absent or very low in all WT tissues, except for the kidney. Renal tubules in these mice were Gb_3_ positive. By comparison, Gb_3_ staining was markedly elevated in all Fabry tissues. In the kidney, staining extended beyond tubules and included glomeruli. Aside from the brain, all organs demonstrated tissue-wide staining of Gb_3_.

MF tissue Gb_3_ staining was, for the most part, similar to that in Fabry tissues (data not shown). MF brain and liver appeared to show less Gb_3_ staining than the corresponding Fabry tissues, but MCD treatment increased staining to levels comparable in MCD-treated Fabry sections. MF lung appeared to show increased staining. While the spleens of both Fabry and MF mice appeared to show a global distribution of Gb_3_, white pulp regions of that tissue demonstrated less signal.

## Discussion

Fabry mice, characterized by a knockout in the *GLA* gene, were generated two decades ago [10]. Although the Fabry mouse suffers from substantial accumulation of Gb_3_, it does not display an overt phenotype and, therefore, does not recapitulate well the clinical course of the human disease. The reasons behind the lack of a pronounced phenotype are unclear, but it is possible that mice express a gene that is protective against the effects of Gb_3_ storage, that there is a limited accumulation of Gb_3_ in the Fabry mouse as has previously been suggested [53], or that the mouse tissue Gb_3_ species profile is distinct from humans. To date, there have been very limited studies on the Fabry mouse Gb_3_ acyl chain species, let alone the rest of the glycosphingolipidome. The advent of new chromatographic and detection technologies with enhanced sensitivities is making acyl chain characterization the standard of analysis in sphingolipid biology. Thus, in an effort to thoroughly characterize the specific GSL acyl chain species expression profile in Fabry mouse tissues, we have performed detailed MS analyses of Gb_3_ as well as its neutral sphingolipid precursors.

This study is, to our knowledge, the first in-depth characterization of multiple GSL acyl chain species in multiple tissues of the Fabry mouse. A study of only Gb_3_ and DHC acyl chain species was recently reported, but the analyses in that study were limited to the kidney and utilized a different *Gla*-deficient mouse [54]. Another study has shown sex differences of Gb_3_ species levels in Fabry and WT mouse kidney and urine [55]. Here, we first show by detailed MS analyses of Gb_3_ that a distinct tissue-specific distribution of Gb_3_ species exists in WT mice. The brain and heart contained only a small subset of Gb_3_ species, while the lung and spleen exhibited the most diverse Gb_3_ profiles. The reasons for the varying degrees of heterogeneity in Gb_3_ expression are unclear but hint toward yet to be determined complex tissue-specific functional roles. While this may be true, retaining homeostatic levels of each species may not be essential, at least in the Fabry mouse. The effects of α-gal A knockout on mouse tissue Gb_3_ levels are of considerable interest since the detection (i.e., accumulation) of particular Gb_3_ species in the Fabry mouse, which are absent in the WT, suggests that these species are, in fact, synthesized in WT mice but are rapidly degraded. This raises questions as to the homeostatic control of differential tissue and cellular GSL expression at large and the possible widespread function of transitory Gb_3_. Furthermore, it is possible that the acyl chain distribution of Fabry mouse tissue Gb_3_ is unique from that in Fabry disease patients. In addition, the shift in percentage composition of individual species may prove to be crucial. Since GSLs are known to be part of membrane microdomains [56–58], a shift in the composition of these microdomains could impact signaling through receptors that are known to function within these domains [59–61].

Identifying biomarkers of Fabry disease has proven difficult, but it has been shown that disease severity does not necessarily correlate with Gb_3_ content [5]. These results, however, refer to total Gb_3_. Indeed differences in urinary Gb_3_ species have been shown to have clinical utility as diagnostic approaches, including for the diagnosis of women with Fabry disease [62,63]. In the present study, we have shown that varying degrees of Gb_3_ species accumulate, ranging from no increase of some species in tissues from Fabry mice to greater than 1000-fold elevations in others. Species that are only modestly elevated in Fabry tissues – as well as those that remain undetectable – might be under tight regulation so as to retain these species at near homoeostatic levels. Identifying these regulatory mechanisms is of key interest to understanding the complex – and unique – behaviors of GSLs. It is indeed possible that the Fabry mouse has mechanisms to maintain these species under tight regulation, while human patients may lack such control systems. The degree to which a specific species is changed is also unique to each tissue. Thus, Fabry disease pathology may correlate to an array of specific Gb_3_ species depending on the affected tissue, and the severity of disease may arise in part from a differential capacity of specific mutant forms of α-gal A to catabolize these species.

α-gal A depletion had a variable effect on tissue DHCs, with some species being elevated, some reduced and most unchanged. The greatest number of significant changes was seen in the kidney, with five of the six species being elevated. A recent study has also shown increases in kidney DHCs in the Fabry mouse [54]. The authors of that study state that galabiosylceramides comprise the majority of DHCs in the kidney. These gala-series GSLs are, in fact, expected to be elevated in Fabry disease, as they also contain the terminal Gal(α 1→4)Gal carbohydrate chain that is recognized by α-gal A. While galabiosylceramides may contribute to the DHC measurements in kidney, this is unlikely the case in other tissues, given that DHC storage was limited in these tissues. However, it is indeed possible that the specific DHC species that were elevated correspond to galabiosylceramides and not lactosylceramides. It is evident that separating these two GSLs (as well as galactosylceramide [GalCer] and GlcCer in the MHCs) would help clarify these analyses.

Surprisingly, the accumulating substrate in the Fabry mouse is not limited to Gb_3_. Instead, all MHC species were markedly elevated. Unlike the case for Gb_3_, however, within a tissue, each MHC species was increased by a similar degree. The exception to this was the Fabry mouse heart, in which C24:1 MHC showed a larger fold-increase than the other species (Figure 3 & Table 3). While MHCs consist of both GlcCer and GalCer, the latter is primarily found in the brain. Measurement of MHCs, therefore, likely refers predominantly to GlcCer. Correspondingly, Fabry mouse brain tissues showed the lowest fold-change in MHCs. The accumulation of MHCs is particularly intriguing given that levels of most Fabry mouse DHCs were unchanged (and some even decreased) in comparison to WT. A previous study looking at renal GSLs showed MHC species to generally be unchanged in the Fabry mouse kidney; however, as mentioned, this study used a different Fabry mouse model and performed analyses at 70 weeks age [54].

It is possible that the increases in MHCs we observed are contributed by downregulation of glucocerebrosidase, the enzyme responsible for GlcCer hydrolysis. This might also explain why the fold elevations are similar among most species. To this end, qPCR analysis of mRNA levels of *Gba1*, the gene encoding glucocerebrosidase, revealed levels to be unchanged in the Fabry liver relative to WT, but the nonlysosomal glucosylceramidase, *Gba2*, was modestly (not significantly) reduced. GlcCer synthase (*Ugcg*) mRNA levels were unaltered, suggesting that the elevation in MHCs is not due to increased synthesis of the lipid but may instead be caused by decreased cytosolic catabolism. Transcript levels of *B4Galt5*, one of the two LacCer synthases [64], were also reduced in the Fabry liver, a possible consequence of a negative feedback response to limit Gb_3_ precursor (i.e., LacCer) availability. However, given the substantial increase in MHCs seen in the Fabry mouse tissues, LacCer synthases would be expected to be elevated. It appears, therefore, that precursor levels and downstream product accumulation may both impact LacCer synthase transcript levels.

In addition, we showed mRNA levels of FAPP2, a protein known to be involved in the cytosolic transfer of GlcCer between organelles [50,51], to be markedly downregulated. Since both GBA2 and FAPP2 act on cytosolic GlcCer, their levels may be linked such that downregulation of FAPP2 is sensed by the cell, which responds by decreasing GBA2 to elevate cytosolic-oriented GlcCer levels for access to FAPP2. Importantly, FAPP2 depletion has been reported to selectively decrease cellular Gb_3_ content [65]; thus, a reduction of this protein in the Fabry mouse, at least in the liver, may in fact be limiting the observed Gb_3_ storage in the mouse.

An interesting finding was the number of strong correlations, both positive and negative, between many of the sphingolipid species within and across tissues. Surprisingly, very few of these were significantly different between WT and Fabry mice, suggesting strong regulatory mechanisms to maintain appropriate proportions of individual species. One might expect that similar acyl chains of a lipid may show better correlations than, for instance, a short chain with a long chain or a saturated with an unsaturated acyl chain; however, no such trend was noticeable. Many species showed strong correlations with all species of a given tissue, while some showed sporadic correlation patterns and yet others showed no strong correlations. Interestingly, several species showed strong negative correlations with species from other tissues, such as those between brain and lung Gb_3_ species or between Fabry heart and kidney MHCs. Moreover, very few WT sphingolipid species showed significant correlations between lipid class, while Fabry species showed a greater number of such correlations. Among these, brain correlations were the most significantly different between Fabry and WT, with WT brain ceramides negatively correlating with brain MHCs. These findings bring to rise questions as to how sphingolipid species levels are regulated in the normal and diseased states, and whether the mechanisms involved transcend the local cellular – and even tissue – levels of regulation.

We postulated that any accumulations in MHCs, DHCs and Gb_3_ could potentially be addressed through an innovative SRT approach that targets ABCB1. We have previously suggested that ABCB1 plays a key role in cellular GSL biosynthesis by mediating the translocation of GlcCer from its site of synthesis on the cytosolic leaflet of the Golgi to the luminal leaflet for access to downstream glycosyltransferases [26,33,35]. Surprisingly, ABCB1 depletion did not reduce Gb_3_ levels; rather, several species were actually increased, particularly in the liver and spleen of MF mice. We hypothesized that knockout of ABCB1 may instead have a predominant effect on another branch of GSL synthesis, namely the gangliosides. With the exception of two species in the heart, however, no significant changes were seen in GM3, GM2 and GM1 between Fabry and MF tissues (Supplementary Figure 3).

We next examined whether ABCB1 deletion affected levels of those GSLs directly involved in the proposed GSL flippase function of ABCB1: GlcCer and LacCer. Separate pools of these GSLs for neutral versus acidic GSL synthesis have been proposed [51,66] and two distinct enzymes have been identified to synthesize LacCer, B4GalT5 and B4GalT6 [64]. Strangely, any changes that were observed were elevations in DHCs and reductions in MHCs. A recent study showed ABCB1 expression to positively correlate to B4GalT5 expression [67]. While it is unknown whether the converse is true, it is possible that the absence of ABCB1 causes a decrease in B4GalT5, in line with our qPCR data.

The role of ABCB1 in GSL metabolism is obviously far more complex than initially thought. ABCB1 is a membrane protein that is found within the same microdomain as some GSL metabolic enzymes and the intimate relationship with GSLs and their metabolic enzymes, particularly GlcCer synthase, is well documented [68–76]. The observed reduction of Gb_3_ using cyclosporine A to treat Fabry mice from our earlier study may be a consequence of the off-target inhibitory effects of the drug. While it is an ABCB1 substrate, cyclosporine A is known to be nonspecific. We also cannot rule out the possibility that knockout of ABCB1 is compensated by overexpression of another flippase or GlcCer transport protein in mice that is evidently capable of completely compensating for ABCB1 loss. In this way, the effect of ABCB1 on GSL synthesis is being confounded by a compensatory mechanism. One candidate flippase is ABCA12, an essential protein responsible for flipping GlcCer into the lamellar granules of the epidermis in skin [77]. This protein is indeed expressed in the Golgi, as is ABCB1 [32,77]. Alternatively, cytosolic GlcCer transport (but not translocase) activity has recently been described for the Golgi-associated FAPP2, both *in vitro* and *in vivo* [50,51].

## Conclusion & future perspective

We have shown that α-gal A deficiency in the Fabry mouse leads to differential storage of individual Gb_3_ species within a tissue, as well as varying degrees of accumulation of the same species across tissues. In addition, α-gal A deficiency has effects beyond a systemic storage of its Gb_3_ substrate; there is also storage of MHCs in all tissues and varying effects on tissue DHCs. Although each of the sphingolipid acyl chain species shows complex correlation patterns, they are generally retained between WT and Fabry mice. How individual GSL species are independently regulated is a key question that remains to be answered. It is clear, however, that acquiring an understanding of the functional roles of acyl chain species of sphingolipids is a necessary step to better understand disease pathogenesis.

The data presented in this study indicate that an intricate relationship exists between specific sphingolipid acyl chain species of the same lipid type within and across tissues. Moreover, specific sphingolipid relationships are altered in the pathological state. Such extensive analyses have the potential to enable identification of highly specific biomarkers of pathologies – or even particular clinical symptoms – in which sphingolipids are implicated. Since Fabry disease patients may suffer from a variety of different symptoms that do not seem to correlate directly with total Gb_3_ content, a closer examination of the tissue profiles of specific acyl chain species of Gb_3_ – and other GSLs, most notably the MHCs – is necessary. With big-data studies becoming more common, we anticipate that detailed correlational analyses will become routine for biochemical data acquired from patient samples. Performing correlation analyses similar to those done in this study may hint toward a unique subset of specific sphingolipids that is dependent on clinical presentation. Based on these potential markers, therapeutic strategies would then be devised to ‘normalize’ their expression. Current therapeutic approaches that ameliorate symptoms in certain tissues may, in fact, be altering specific sphingolipid correlations in a beneficial manner, but may not affect other tissues in which a distinct correlation profile is observed. Based on the type of data we have generated, such an outcome may be easier to predict. Thus, the interplay between identified species of interest and other tissue outcomes – and even other pathologies in which sphingolipids may or may not have a defined contribution – will be better understood. Finally, from a biological perspective, by understanding the relationships between particular sphingolipids, correlational analyses may provide insights into the mechanisms regulating expression of sphingolipids, an arena that is at present poorly understood.

**Table 1.** **Tissue globotriaosylceramide species percent composition.**

	**Brain**		**Heart**		**Kidney**		**Liver**		**Lung**		**Spleen**	
**Acyl chain**	**WT**	**Fabry**	**WT**	**Fabry**	**WT**	**Fabry**	**WT**	**Fabry**	**WT**	**Fabry**	**WT**	**Fabry**
C14:0	ND	ND	ND	ND	ND	ND	ND	0.1 ± 0.0	ND	ND	ND	0.0 ± 0.0
C16:0	17.2 ± 3.6	24.3 ± 17.3	3.7 ± 7.5	6.2 ± 0.9	15.7 ± 9.4	14.2 ± 2.9	2.0 ± 4.0	13.2 ± 8.2	10.9 ± 4.1	9.2 ± 4.5	11.2 ± 5.4	8.5 ± 1.9
C18:0	ND	10.2 ± 3.6	ND	3.5 ± 0.4	0.1 ± 0.1	2.1 ± 0.2	ND	4.8 ± 3.2	3.2 ± 1.1	4.9 ± 2.0	3.6 ± 1.4	4.7 ± 1.0
C20:0	ND	8.2 ± 1.1	ND	8.8 ± 0.5	1.6 ± 0.5	10.5 ± 1.7	2.0 ± 4.0	5.9 ± 3.2	11.5 ± 5.0	11.5 ± 3.7	11.4 ± 3.1	8.9 ± 1.5
C22:0	ND	10.0 ± 2.1	28.4 ± 22.4	17.0 ± 2.0	16.2 ± 7.0	14.5 ± 1.0	27.4 ± 19.8	14.9 ± 5.3	22.3 ± 8.6	16.9 ± 2.4	20.7 ± 3.3	10.4 ± 0.5
C24:0	ND	9.8 ± 4.2	21.7 ± 15.4	20.6 ± 3.6	21.7 ± 10.7	22.8 ± 1.4	20.4 ± 11.8	17.1 ± 7.7	13.3 ± 4.5	15.5 ± 3.1	12.3 ± 1.7	19.9 ± 2.0
C26:0	ND	1.0 ± 0.7	ND	0.4 ± 0.1	ND	0.6 ± 0.0	ND	0.6 ± 0.2	1.0 ± 0.7	0.9 ± 0.4	ND	0.5 ± 0.1
C16:1	62.2 ± 2.1	5.0 ± 0.3	23.5 ± 2.6	0.2 ± 0.0	6.2 ± 2.8	2.7 ± 0.7	19.9 ± 4.3	1.5 ± 0.6	3.0 ± 0.3	0.6 ± 0.1	4.1 ± 0.7	0.5 ± 0.0
C18:1	ND	1.3 ± 0.6	ND	0.2 ± 0.0	ND	0.2 ± 0.0	ND	0.6 ± 0.3	0.3 ± 0.3	0.2 ± 0.1	0.2 ± 0.2	0.3 ± 0.1
C20:1	ND	2.2 ± 0.5	ND	0.7 ± 0.1	0.1 ± 0.2	0.7 ± 0.2	ND	1.0 ± 0.8	ND	0.6 ± 0.2	0.3 ± 0.6	0.7 ± 0.1
C22:1	1.0 ± 2.0	1.7 ± 0.5	ND	2.2 ± 0.3	4.2 ± 3.3	2.8 ± 1.1	ND	1.6 ± 0.8	2.5 ± 1.0	2.7 ± 0.9	2.2 ± 1.5	2.4 ± 0.3
C24:1	ND	10.2 ± 1.2	19.7 ± 11.2	18.9 ± 1.9	15.7 ± 6.8	13.5 ± 0.8	18.9 ± 13.9	18.6 ± 8.2	12.2 ± 2.5	14.0 ± 2.6	17.0 ± 2.3	17.3 ± 1.4
C24:2	ND	5.1 ± 1.2	ND	4.5 ± 0.6	2.3 ± 1.1	2.7 ± 0.2	ND	5.1 ± 3.2	2.6 ± 1.8	4.6 ± 2.1	5.7 ± 3.2	9.2 ± 1.4
C26:1	ND	0.6 ± 0.3	ND	0.2 ± 0.0	0.0 ± 0.1	0.1 ± 0.0	ND	0.2 ± 0.1	ND	0.3 ± 0.1	ND	0.1 ± 0.0
C22:2	ND	0.3 ± 0.1	ND	0.2 ± 0.0	ND	0.1 ± 0.0	ND	0.6 ± 0.4	0.8 ± 0.6	1.1 ± 0.5	ND	0.4 ± 0.1
C24:1OH	0.7 ± 1.4	7.1 ± 0.9	ND	0.6 ± 0.0	6.9 ± 2.3	2.9 ± 0.4	ND	1.1 ± 0.6	3.2 ± 1.3	2.4 ± 0.8	0.5 ± 0.4	0.6 ± 0.1
C14:0Me	ND	0.3 ± 0.2	ND	0.0 ± 0.0	ND	0.2 ± 0.0	ND	0.3 ± 0.2	ND	0.1 ± 0.1	ND	0.2 ± 0.0
C16:0Me	6.3 ± 1.8	0.7 ± 0.3	2.8 ± 5.7	0.2 ± 0.0	0.1 ± 0.1	0.1 ± 0.0	1.2 ± 2.4	0.4 ± 0.2	0.5 ± 0.4	0.2 ± 0.1	0.5 ± 0.4	0.2 ± 0.0
C18:0Me	ND	0.3 ± 0.1	ND	1.1 ± 0.1	ND	0.6 ± 0.1	ND	1.0 ± 0.7	0.4 ± 0.5	0.8 ± 0.3	0.4 ± 0.5	0.9 ± 0.2
C20:0Me	ND	0.4 ± 0.1	ND	2.1 ± 0.2	0.2 ± 0.2	0.8 ± 0.1	ND	1.5 ± 0.6	2.5 ± 0.8	2.7 ± 0.7	1.8 ± 0.3	1.8 ± 0.1
C22:1Me	12.6 ± 4.1	0.7 ± 0.2	ND	1.0 ± 0.1	1.4 ± 1.1	0.7 ± 0.2	ND	1.0 ± 0.6	ND	0.5 ± 0.1	ND	1.1 ± 0.1
C22:0Me	ND	0.7 ± 0.3	ND	9.7 ± 0.9	6.2 ± 3.6	5.1 ± 0.7	8.3 ± 6.8	7.5 ± 3.6	8.0 ± 3.0	8.1 ± 1.9	7.3 ± 1.0	8.8 ± 0.8
C24:0Me	ND	ND	ND	1.4 ± 0.2	1.5 ± 0.8	1.6 ± 0.2	ND	1.3 ± 0.7	1.8 ± 1.2	2.0 ± 0.7	0.8 ± 0.2	2.2 ± 0.2
C24:1Me	ND	ND	ND	0.3 ± 0.0	ND	ND	ND	0.2 ± 0.1	ND	0.2 ± 0.1	ND	0.2 ± 0.0

The fractional proportion of each globotriaosylceramide species assessed is represented as a percentage of total globotriaosylceramide measured ± standard deviation (n = 4).

ND: Not detected; WT: Wild-type.

**Table 2.** **MF mouse tissue glycosphingolipid fold-change relative to Fabry.**

	**Brain**			**Heart**			**Kidney**			**Liver**			**Lung**			**Spleen**		
**Acyl chain**	**Gb_3_**	**DHC**	**MHC**	**Gb_3_**	**DHC**	**MHC**	**Gb_3_**	**DHC**	**MHC**	**Gb_3_**	**DHC**	**MHC**	**Gb_3_**	**DHC**	**MHC**	**Gb_3_**	**DHC**	**MHC**
C14:0										1.7						1.6		
C16:0	1.1	1.2	0.9	1.0	1.1	0.4	0.9	1.4	0.9	1.7	1.8	0.7	0.8	1.1	0.8	1.3	1.4	0.6
C18:0	1.3	1.0	0.9	0.9	1.6	0.5	1.0	1.2	0.6	1.8	1.1	0.7	0.8	1.4	0.7	1.1	1.5	0.5
C20:0	1.1	0.7	0.9	0.9	1.5	0.6	0.9	1.1	0.6	1.6	0.9	0.7	0.8	1.5	0.7	1.1	1.4	0.5
C22:0	0.9	0.8	0.8	1.0	1.3	0.5	1.0	1.2	0.7	1.2	0.6	0.8	0.9	1.3	0.7	1.2	1.4	0.6
C24:0	0.8	1.0	0.9	1.0	1.9	0.3	1.1	1.2	0.8	1.5	1.1	1.0	0.9	1.8	1.0	1.2	1.7	0.8
C26:0	0.7			1.0			1.1			1.7			0.8			1.2		
C16:1	0.9			1.0			0.8			1.8			1.0			1.2		
C18:1	1.1			1.1			0.9			1.8			1.3			1.3		
C20:1	1.1			0.8			0.7			1.5			0.9			1.3		
C22:1	1.0			0.9			0.9			1.7			0.7			1.1		
C24:1	0.9	1.1	1.0	1.0	1.0	0.5	1.1	1.2	0.7	1.4	0.7	1.0	1.0	1.1	0.8	1.2	1.5	0.7
C24:2	0.9			1.1			1.2			2.4			0.9			1.4		
C26:1	1.2			1.1			1.3			1.9			1.0			1.4		
C22:2	0.8			1.0			1.2			1.8			0.6			1.5		
C24:1OH	1.1			1.1			0.8			1.6			0.7			1.2		
C14:0Me	0.9			0.8			0.8			2.4			0.6			1.5		
C16:0Me	0.9			1.2			1.0			2.0			1.0			1.4		
C18:0Me	1.0			0.8			0.9			1.7			0.8			1.3		
C20:0Me	0.9			0.9			0.9			1.6			0.7			1.0		
C22:1Me	1.3			1.0			0.8			2.2			0.9			1.1		
C22:0Me	0.7			1.0			1.0			1.7			0.9			1.2		
C24:0Me				1.1			1.0			2.0			0.8			1.3		
C24:1Me				1.1						2.3			1.1			1.4		

DHC: Dihexosylceramide; Gb_3_: Globotriaosylceramide; MHC: Monohexosylceramide.

**Table 3.** **Fabry mouse tissue sphingolipid fold-change relative to wild-type.**

	**Brain**			**Heart**			**Kidney**			**Liver**			**Lung**			**Spleen**		
**Acyl chain**	**Cer**	**MHC**	**DHC**	**Cer**	**MHC**	**DHC**	**Cer**	**MHC**	**DHC**	**Cer**	**MHC**	**DHC**	**Cer**	**MHC**	**DHC**	**Cer**	**MHC**	**DHC**
C16:0	1.27	1.90	0.60	1.16	7.31	1.62	0.97	2.06	0.62	1.24	3.53	1.69	0.61	3.27	0.68	0.41	5.99	0.70
C18:0	1.02	2.86	1.88	0.71	18.99	0.81	1.08	3.25	1.92	0.80	6.17	2.12	0.65	8.39	1.66	0.40	11.05	1.05
C20:0	1.01	2.24	1.40	0.80	8.72	0.92	1.15	4.22	2.15	0.96	5.79	1.92	0.77	4.89	0.99	0.46	5.97	0.85
C22:0	1.10	2.08	1.05	0.88	10.36	1.04	1.27	4.93	2.27	0.98	5.44	1.61	0.79	5.16	1.05	0.53	5.92	0.85
C24:0	1.33	1.27	0.44	1.22	17.75	0.79	1.05	3.27	2.37	1.00	3.78	1.56	0.88	3.42	1.68	0.67	4.97	0.56
C24:1	1.10	2.46	1.44	1.53	37.22	2.11	1.30	3.56	4.27	1.28	4.76	6.90	1.02	4.86	2.65	0.67	6.59	2.57

Cer: Ceramide; DHC: Dihexosylceramide; MHC: Monohexosylceramide.

**Table 4.** **Tissue dihexosylceramide species percent composition.**

	**Brain**		**Heart**		**Kidney**		**Liver**		**Lung**		**Spleen**	
**Acyl chain**	**WT**	**Fabry**	**WT**	**Fabry**	**WT**	**Fabry**	**WT**	**Fabry**	**WT**	**Fabry**	**WT**	**Fabry**
C16:0	8.9 ± 1.3	3.3 ± 0.8	8.9 ± 4.1	14.2 ± 2.5	74.8 ± 27.6	41.5 ± 11.7	30.1 ± 12.0	26.0 ± 14.1	48.8 ± 20.5	29.8 ± 13.1	32.8 ± 3.9	26.4 ± 10.0
C18:0	74.3 ± 9.1	86.7 ± 11.3	13.5 ± 2.6	10.8 ± 3.4	0.6 ± 0.2	1.1 ± 0.2	2.1 ± 1.3	2.2 ± 1.0	4.6 ± 0.8	6.8 ± 1.9	7.7 ± 0.9	9.3 ± 1.7
C20:0	3.3 ± 0.7	2.9 ± 1.8	42.2 ± 6.8	38.1 ± 3.9	1.1 ± 0.2	2.1 ± 0.7	4.1 ± 1.2	4.0 ± 2.6	9.6 ± 2.3	8.5 ± 2.8	13.8 ± 2.0	13.6 ± 4.3
C22:0	1.4 ± 0.1	0.9 ± 0.3	19.8 ± 5.4	20.3 ± 2.7	4.4 ± 1.3	9.0 ± 1.1	27.6 ± 17.8	22.8 ± 23.0	13.0 ± 4.9	12.2 ± 4.2	17.0 ± 1.6	16.7 ± 3.6
C24:0	7.4 ± 7.4	2.0 ± 0.3	12.2 ± 4.2	9.4 ± 2.1	15.5 ± 4.1	32.9 ± 6.9	30.3 ± 13.0	24.3 ± 24.2	16.5 ± 5.7	24.8 ± 6.6	22.0 ± 1.5	14.2 ± 4.5
C24:1	4.6 ± 0.9	4.1 ± 1.1	3.4 ± 0.6	7.1 ± 1.2	3.5 ± 1.0	13.5 ± 4.3	5.8 ± 1.8	20.7 ± 18.9	7.5 ± 2.3	17.9 ± 7.5	6.6 ± 2.2	19.8 ± 6.0

The fractional proportion of each DHC species assessed is represented as a percentage of total dihexosylceramide measured ± standard deviation (n = 4–5).

WT: Wild-type.

**Table 5.** **Tissue monohexosylceramide species percent composition.**

**Acyl chain**	**Brain**		**Heart**		**Kidney**		**Liver**		**Lung**		**Spleen**	
	**WT**	**Fabry**	**WT**	**Fabry**	**WT**	**Fabry**	**WT**	**Fabry**	**WT**	**Fabry**	**WT**	**Fabry**
C16:0	1.0 ± 0.1	0.9 ± 0.1	13.8 ± 8.6	6.5 ± 3.7	40.0 ± 11.8	27.7 ± 9.7	23.2 ± 5.5	19.0 ± 6.1	23.1 ± 7.6	19.6 ± 8.3	16.1 ± 6.0	17.0 ± 5.8
C18:0	5.6 ± 1.7	7.7 ± 1.2	3.9 ± 2.6	4.7 ± 4.1	0.6 ± 0.5	0.6 ± 0.2	0.8 ± 0.3	1.2 ± 0.4	1.4 ± 0.5	3.1 ± 1.6	1.7 ± 0.7	3.4 ± 1.4
C20:0	2.9 ± 0.8	3.1 ± 0.6	14.6 ± 6.1	8.2 ± 3.6	2.4 ± 1.4	3.4 ± 1.3	3.4 ± 0.9	4.6 ± 1.8	5.3 ± 2.6	6.8 ± 2.7	6.4 ± 3.5	6.7 ± 2.6
C22:0	8.8 ± 0.9	8.8 ± 1.2	25.1 ± 14.3	16.7 ± 8.2	8.5 ± 3.2	14.1 ± 4.7	23.6 ± 4.8	29.8 ± 10.7	9.9 ± 4.4	13.3 ± 5.4	16.1 ± 7.0	16.7 ± 4.8
C24:0	29.5 ± 6.8	17.9 ± 2.3	30.6 ± 18.7	35.0 ± 35.4	39.9 ± 11.2	43.9 ± 11.9	37.8 ± 6.3	33.1 ± 7.4	50.0 ± 22.8	44.3 ± 14.1	45.0 ± 21.3	39.2 ± 15.8
C24:1	52.2 ± 4.9	61.6 ± 6.4	12.0 ± 4.7	28.9 ± 34.3	8.6 ± 6.0	10.2 ± 2.0	11.1 ± 2.7	12.3 ± 3.8	10.2 ± 5.0	12.9 ± 5.3	14.7 ± 6.0	17.0 ± 6.0

The fractional proportion of each monohexosylceramide species assessed is represented as a percentage of total monohexosylceramide measured ± standard deviation (n = 4–5).

WT: Wild-type.

**Executive summary**

**Background**

Fabry disease is an X-linked lysosomal storage disorder caused by deficient α-galactosidase A activity leading to progressive accumulation of terminal α-galactose-linked glycosphingolipids (GSLs), predominantly globotriaosylceramide (Gb_3_), in many tissues.
Patients suffer from substantial accumulation of Gb_3_, but clinical manifestations do not seem to correlate directly with total Gb_3_ content.
Studies examining tissue distribution of acyl chain species of Gb_3_ and upstream neutral GSLs are lacking.
We have previously shown pharmacological inhibition of ABCB1 to reduce Gb_3_ levels in Fabry mice

**Experimental**

Tissues from 27-week-old wild-type and Fabry mice were harvested and homogenized. Sphingolipids were extracted and subjected to LC-MS, and the relative amount of each acyl chain species was determined.
A novel mouse model was developed by crossbreeding Fabry mice with ABCB1 knockout mice.

**Results**

A thorough characterization of the Fabry mouse GSL profile revealed a unique Gb_3_ species expression profile between individual Fabry mouse tissues as well as a differential storage of species within those tissues.
Storage of GSLs extended beyond Gb_3_, as all Fabry tissues exhibited significant accumulation of each monohexosylceramide species examined.
Dihexosylceramide accumulation was variable in the tissues, either elevated, reduced or unchanged.
MDR/Fabry mouse exhibited a complex, tissue-dependent effect on Gb_3_.
A highly complex network of correlations exists between individual sphingolipid acyl chain species within and across tissues.

**Conclusion**

The specific sphingolipid acyl chain profile of Fabry mice reveals that storage is not limited to Gb_3_ and that all species are not equally affected by α-galactosidase A deficiency.
The results presented here will help us better understand how specific sphingolipid species correlate with one another and how these correlations change in the α-gal A-deficient state, potentially leading to the identification of highly specific biomarkers of Fabry disease pathology and treatment outcomes.
