# Supplementary figures and images for: Glycosphingolipid storage in Fabry mice extends beyond globotriaosylceramide and is affected by ABCB1 depletion
Source: Future Sci OA. 2016 Oct 13;2(4):FSO147. doi: 10.4155/fsoa-2016-0027 (PMC5242178; doi:10.4155/fsoa-2016-0027)

Supplementary Figure 1

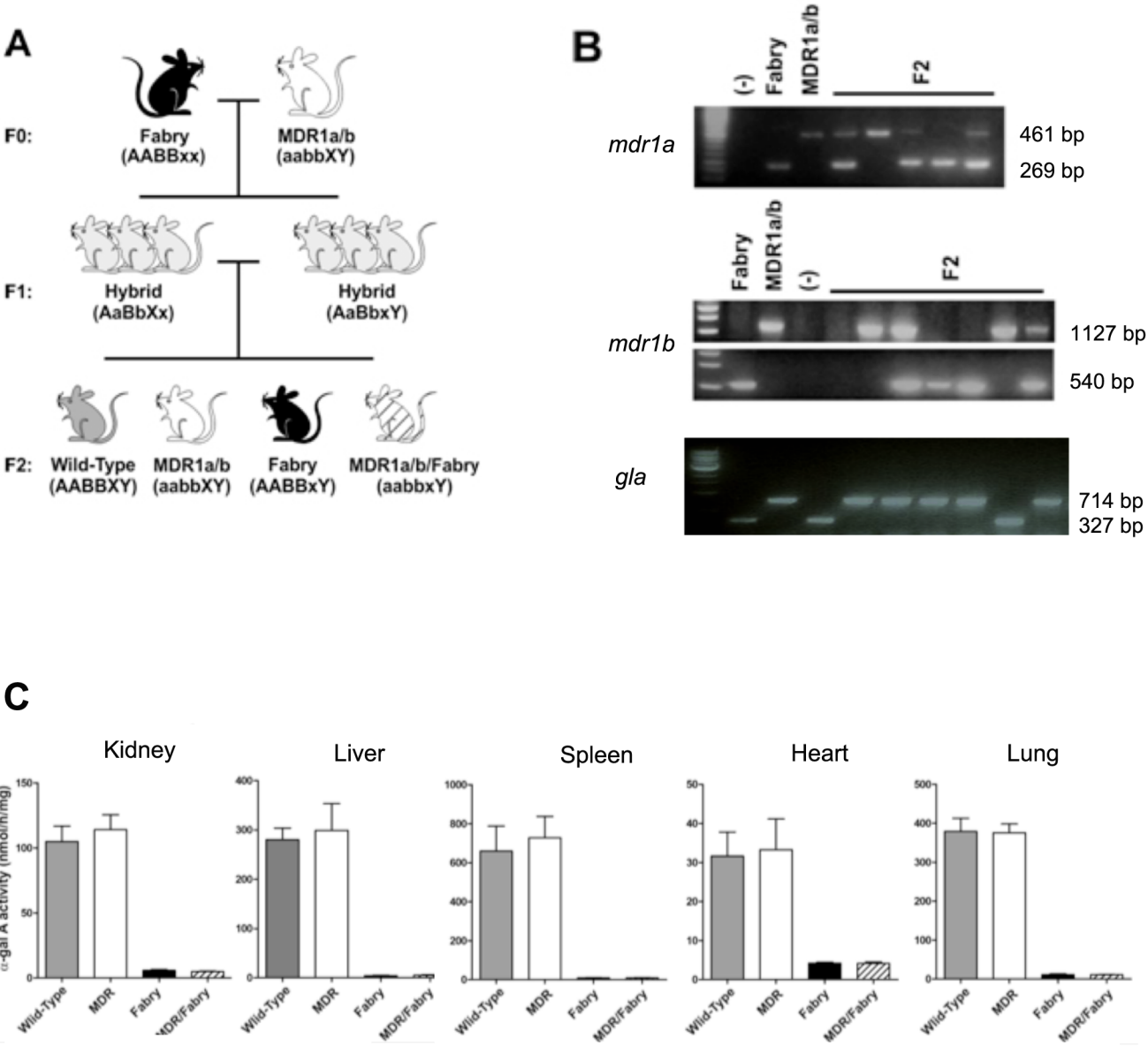

Supplement: Supplementary file 1 [file fsoa-02-147-s1.pdf]

# Supplementary Figure 3

Brain

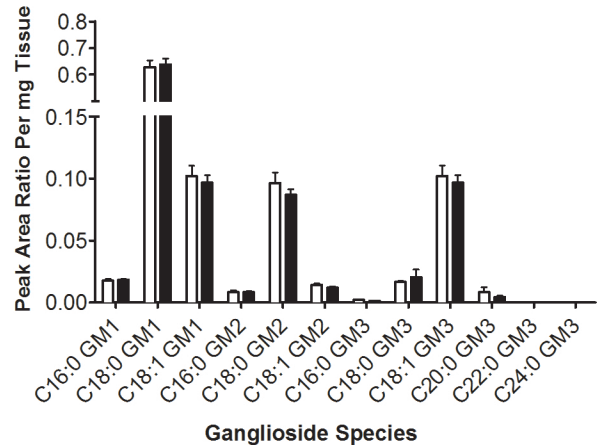

Lung

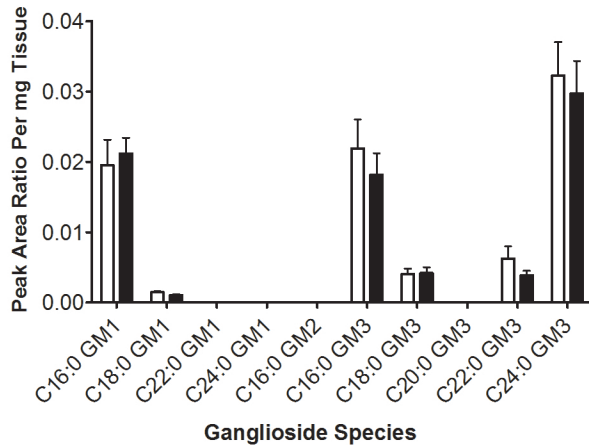

Heart

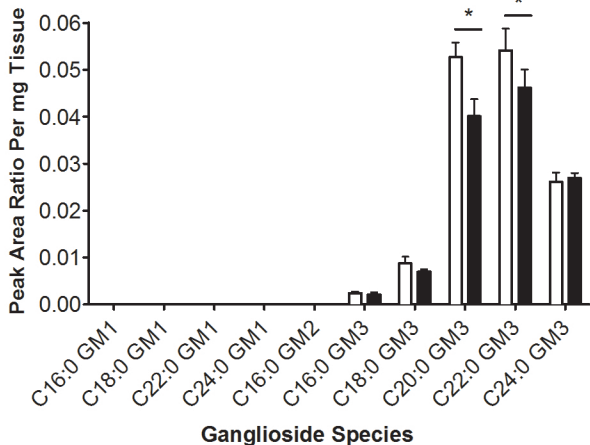

Kidney

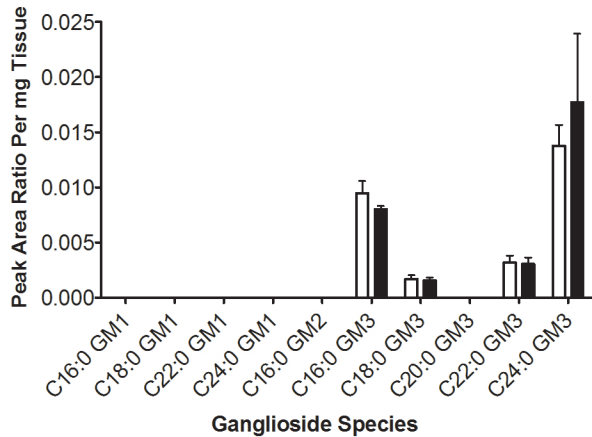

Spleen

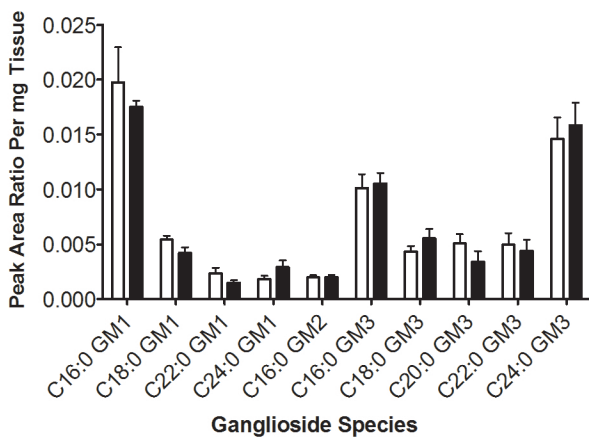

Liver

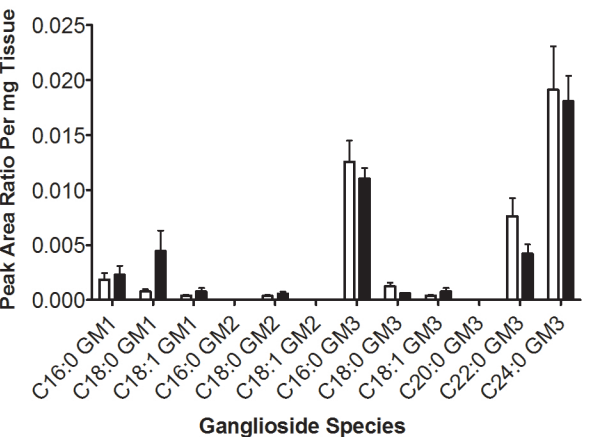

Supplement: Supplementary file 3 [file fsoa-02-147-s3.pdf]
