# Supplementary material for: Glycosphingolipid storage in Fabry mice extends beyond globotriaosylceramide and is affected by ABCB1 depletion
Source: Future Sci OA. 2016 Oct 13;2(4):FSO147. doi: 10.4155/fsoa-2016-0027 (PMC5242178; doi:10.4155/fsoa-2016-0027)
Supplement: Supplementary file 2 [file fsoa-02-147-s2.pdf]

Supplementary Figure 2A

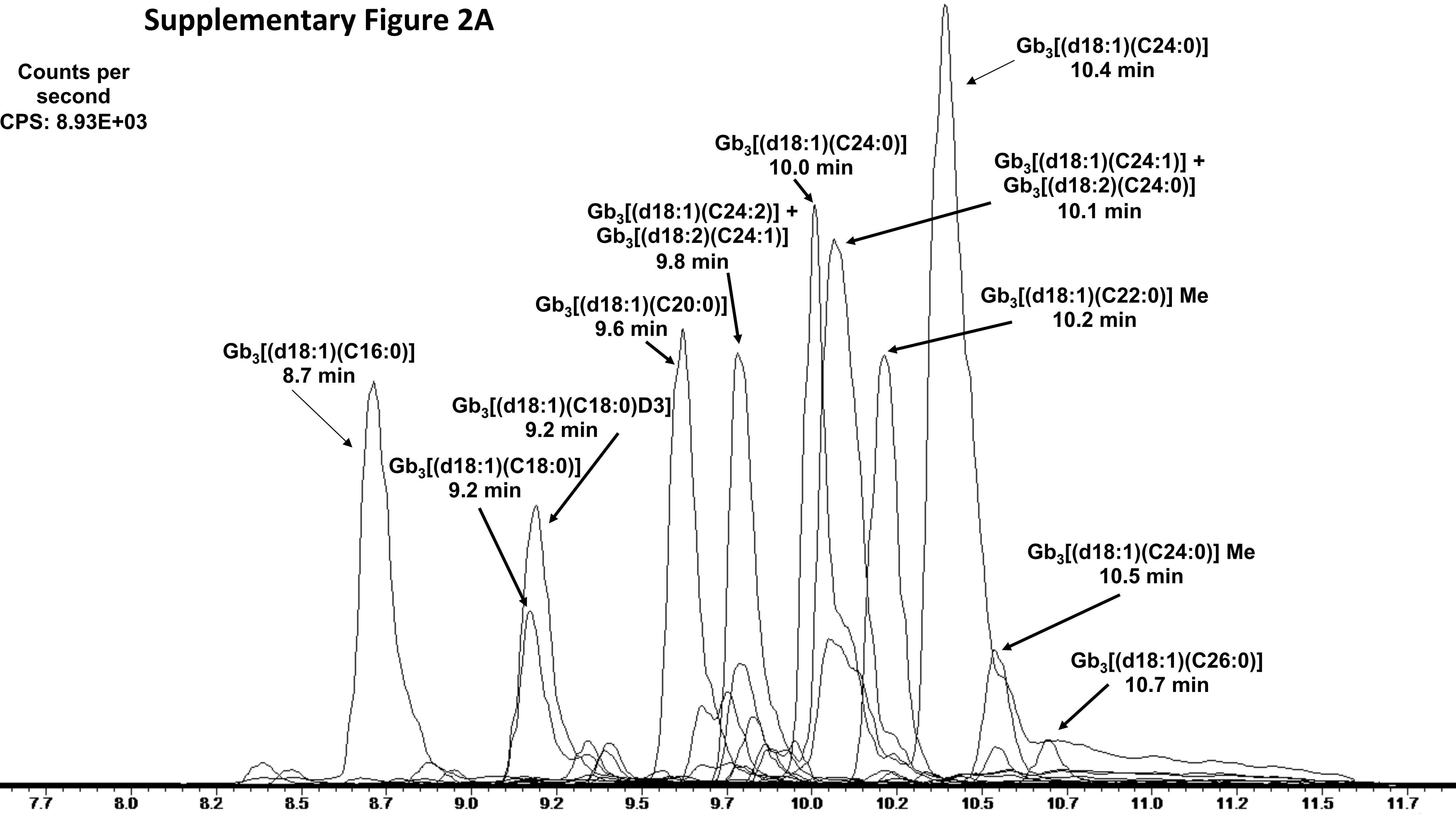

B

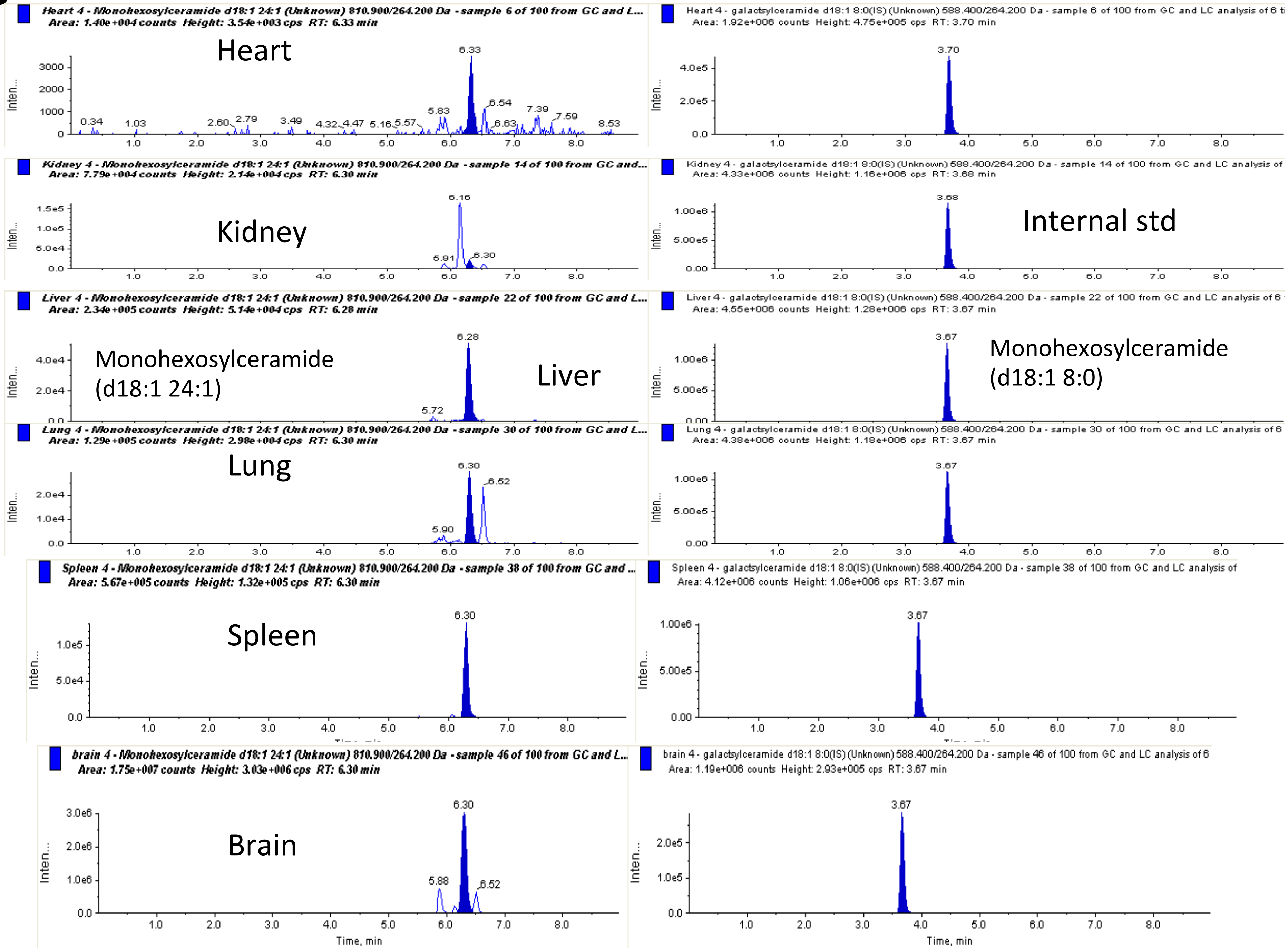
Heart 4 - Monohexosylceramide d18:1 24:1 (Unknown) 810.900/264.200 Da - sample 6 of 100 from GC and L...
Area: 1.40e+004 counts Height: 3.54e+003 cps RT: 6.33 min
Heart
Heart 4 - galactsylceramide d18:1 8:0(IS) (Unknown) 588.400/264.200 Da - sample 6 of 100 from GC and LC analysis of 6 ti
Area: 1.92e+006 counts Height: 4.75e+005 cps RT: 3.70 min
Kidney 4 - Monohexosylceramide d18:1 24:1 (Unknown) 810.900/264.200 Da - sample 14 of 100 from GC and...
Area: 7.79e+004 counts Height: 2.14e+004 cps RT: 6.30 min
Kidney
Kidney 4 - galactsylceramide d18:1 8:0(IS) (Unknown) 588.400/264.200 Da - sample 14 of 100 from GC and LC analysis of
Area: 4.33e+006 counts Height: 1.16e+006 cps RT: 3.68 min
Internal std
Liver 4 - Monohexosylceramide d18:1 24:1 (Unknown) 810.900/264.200 Da - sample 22 of 100 from GC and L...
Area: 2.34e+005 counts Height: 5.14e+004 cps RT: 6.28 min
Monohexosylceramide (d18:1 24:1)
Liver
Liver 4 - galactsylceramide d18:1 8:0(IS) (Unknown) 588.400/264.200 Da - sample 22 of 100 from GC and LC analysis of 6
Area: 4.55e+006 counts Height: 1.28e+006 cps RT: 3.67 min
Monohexosylceramide (d18:1 8:0)
Lung 4 - Monohexosylceramide d18:1 24:1 (Unknown) 810.900/264.200 Da - sample 30 of 100 from GC and L...
Area: 1.29e+005 counts Height: 2.98e+004 cps RT: 6.30 min
Lung
Lung 4 - galactsylceramide d18:1 8:0(IS) (Unknown) 588.400/264.200 Da - sample 30 of 100 from GC and LC analysis of 6
Area: 4.38e+006 counts Height: 1.18e+006 cps RT: 3.67 min
Spleen 4 - Monohexosylceramide d18:1 24:1 (Unknown) 810.900/264.200 Da - sample 38 of 100 from GC and ...
Area: 5.67e+005 counts Height: 1.32e+005 cps RT: 6.30 min
Spleen
Spleen 4 - galactsylceramide d18:1 8:0(IS) (Unknown) 588.400/264.200 Da - sample 38 of 100 from GC and LC analysis of
Area: 4.12e+006 counts Height: 1.06e+006 cps RT: 3.67 min
brain 4 - Monohexosylceramide d18:1 24:1 (Unknown) 810.900/264.200 Da - sample 46 of 100 from GC and L...
Area: 1.75e+007 counts Height: 3.03e+006 cps RT: 6.30 min
Brain
brain 4 - galactsylceramide d18:1 8:0(IS) (Unknown) 588.400/264.200 Da - sample 46 of 100 from GC and LC analysis of 6
Area: 1.19e+006 counts Height: 2.93e+005 cps RT: 3.67 min
Inten...
Time, min

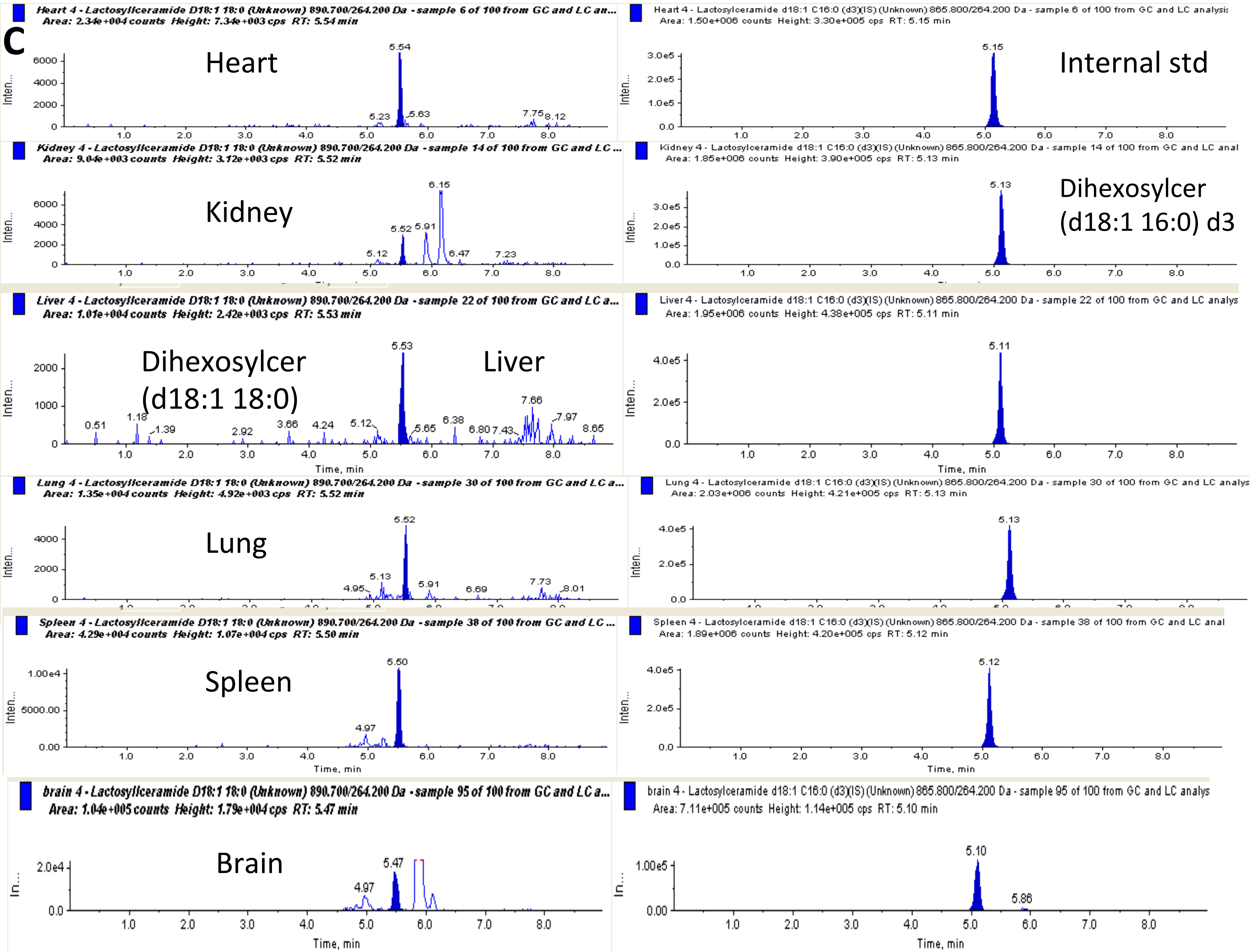
C
Heart 4 - Lactosylceramide D18:1 18:0 (Unknown) 890.700/264.200 Da - sample 6 of 100 from GC and LC an...
Area: 2.34e+004 counts Height: 7.34e+003 cps RT: 5.54 min
Heart
Heart 4 - Lactosylceramide d18:1 C16:0 (d3)(IS) (Unknown) 865.800/264.200 Da - sample 6 of 100 from GC and LC analysis
Area: 1.50e+006 counts Height: 3.30e+005 cps RT: 5.15 min
Internal std
Kidney 4 - Lactosylceramide D18:1 18:0 (Unknown) 890.700/264.200 Da - sample 14 of 100 from GC and LC ...
Area: 9.04e+003 counts Height: 3.12e+003 cps RT: 5.52 min
Kidney
Kidney 4 - Lactosylceramide d18:1 C16:0 (d3)(IS) (Unknown) 865.800/264.200 Da - sample 14 of 100 from GC and LC anal
Area: 1.85e+006 counts Height: 3.90e+005 cps RT: 5.13 min
Dihexosylcer
(d18:1 16:0) d3
Liver 4 - Lactosylceramide D18:1 18:0 (Unknown) 890.700/264.200 Da - sample 22 of 100 from GC and LC a...
Area: 1.01e+004 counts Height: 2.42e+003 cps RT: 5.53 min
Dihexosylcer
(d18:1 18:0)
Liver
Liver 4 - Lactosylceramide d18:1 C16:0 (d3)(IS) (Unknown) 865.800/264.200 Da - sample 22 of 100 from GC and LC analys
Area: 1.95e+006 counts Height: 4.38e+005 cps RT: 5.11 min
Lung 4 - Lactosylceramide D18:1 18:0 (Unknown) 890.700/264.200 Da - sample 30 of 100 from GC and LC a...
Area: 1.35e+004 counts Height: 4.92e+003 cps RT: 5.52 min
Lung
Lung 4 - Lactosylceramide d18:1 C16:0 (d3)(IS) (Unknown) 865.800/264.200 Da - sample 30 of 100 from GC and LC analys
Area: 2.03e+006 counts Height: 4.21e+005 cps RT: 5.13 min
Spleen 4 - Lactosylceramide D18:1 18:0 (Unknown) 890.700/264.200 Da - sample 38 of 100 from GC and LC ...
Area: 4.29e+004 counts Height: 1.07e+004 cps RT: 5.50 min
Spleen
Spleen 4 - Lactosylceramide d18:1 C16:0 (d3)(IS) (Unknown) 865.800/264.200 Da - sample 38 of 100 from GC and LC anal
Area: 1.89e+006 counts Height: 4.20e+005 cps RT: 5.12 min
brain 4 - Lactosylceramide D18:1 18:0 (Unknown) 890.700/264.200 Da - sample 95 of 100 from GC and LC a...
Area: 1.04e+005 counts Height: 1.79e+004 cps RT: 5.47 min
Brain
brain 4 - Lactosylceramide d18:1 C16:0 (d3)(IS) (Unknown) 865.800/264.200 Da - sample 95 of 100 from GC and LC analys
Area: 7.11e+005 counts Height: 1.14e+005 cps RT: 5.10 min
Time, min
Inten...

D

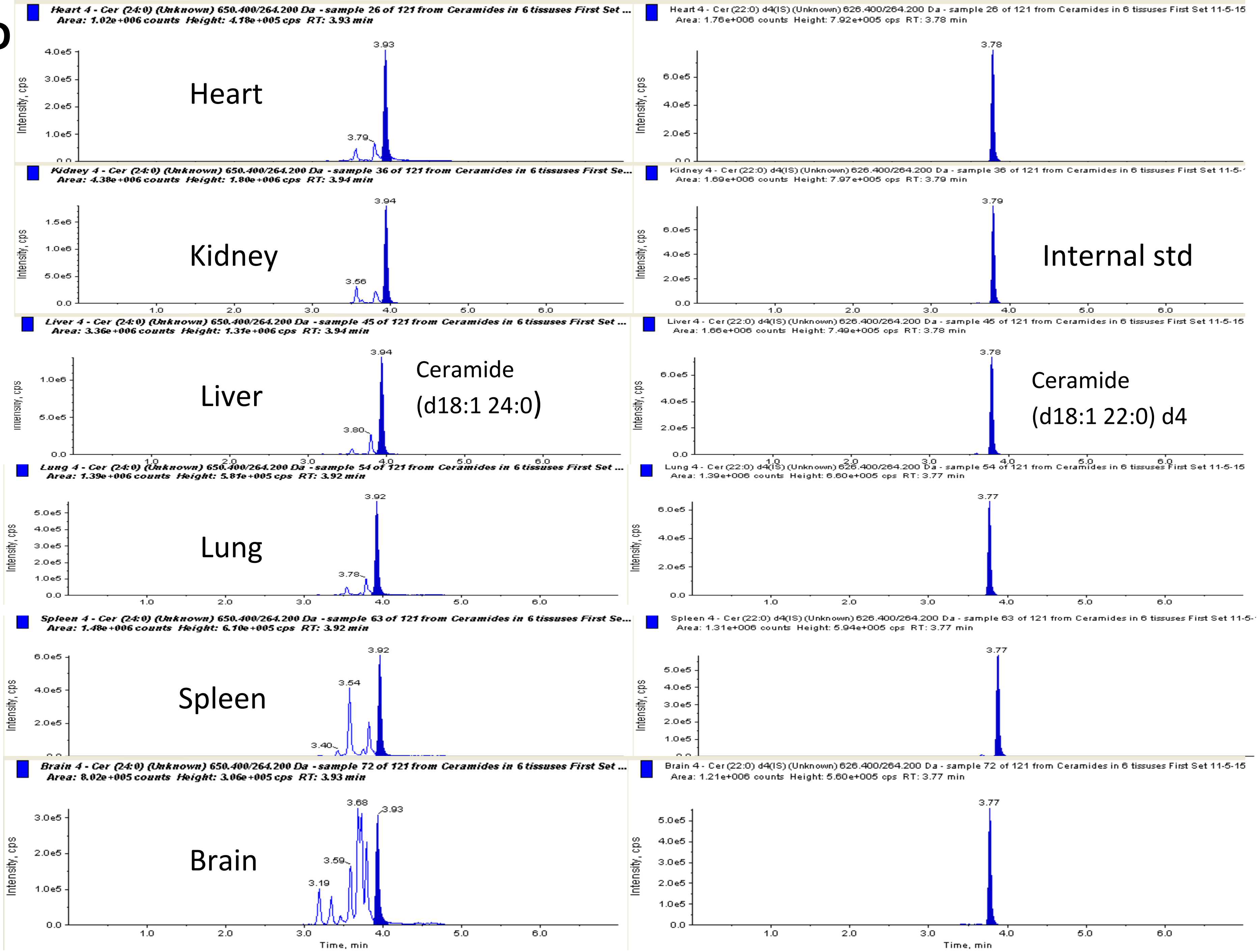
Heart 4 - Cer (24:0) (Unknown) 650.400/264.200 Da - sample 26 of 121 from Ceramides in 6 tissuses First Set ...
Area: 1.02e+006 counts Height: 4.18e+005 cps RT: 3.93 min
Heart
Heart 4 - Cer (22:0) d4(IS) (Unknown) 626.400/264.200 Da - sample 26 of 121 from Ceramides in 6 tissuses First Set 11-5-15
Area: 1.76e+006 counts Height: 7.92e+005 cps RT: 3.78 min
Kidney 4 - Cer (24:0) (Unknown) 650.400/264.200 Da - sample 36 of 121 from Ceramides in 6 tissuses First Se...
Area: 4.38e+006 counts Height: 1.80e+006 cps RT: 3.94 min
Kidney
Kidney 4 - Cer (22:0) d4(IS) (Unknown) 626.400/264.200 Da - sample 36 of 121 from Ceramides in 6 tissuses First Set 11-5-
Area: 1.69e+006 counts Height: 7.97e+005 cps RT: 3.79 min
Internal std
Liver 4 - Cer (24:0) (Unknown) 650.400/264.200 Da - sample 45 of 121 from Ceramides in 6 tissuses First Set ...
Area: 3.36e+006 counts Height: 1.31e+006 cps RT: 3.94 min
Liver
Ceramide (d18:1 24:0)
Liver 4 - Cer (22:0) d4(IS) (Unknown) 626.400/264.200 Da - sample 45 of 121 from Ceramides in 6 tissuses First Set 11-5-15
Area: 1.66e+006 counts Height: 7.49e+005 cps RT: 3.78 min
Ceramide (d18:1 22:0) d4
Lung 4 - Cer (24:0) (Unknown) 650.400/264.200 Da - sample 54 of 121 from Ceramides in 6 tissuses First Set ...
Area: 1.39e+006 counts Height: 5.81e+005 cps RT: 3.92 min
Lung
Lung 4 - Cer (22:0) d4(IS) (Unknown) 626.400/264.200 Da - sample 54 of 121 from Ceramides in 6 tissuses First Set 11-5-15
Area: 1.39e+006 counts Height: 6.60e+005 cps RT: 3.77 min
Spleen 4 - Cer (24:0) (Unknown) 650.400/264.200 Da - sample 63 of 121 from Ceramides in 6 tissuses First Se...
Area: 1.48e+006 counts Height: 6.10e+005 cps RT: 3.92 min
Spleen
Spleen 4 - Cer (22:0) d4(IS) (Unknown) 626.400/264.200 Da - sample 63 of 121 from Ceramides in 6 tissuses First Set 11-5-
Area: 1.31e+006 counts Height: 5.94e+005 cps RT: 3.77 min
Brain 4 - Cer (24:0) (Unknown) 650.400/264.200 Da - sample 72 of 121 from Ceramides in 6 tissuses First Set ...
Area: 8.02e+005 counts Height: 3.06e+005 cps RT: 3.93 min
Brain
Brain 4 - Cer (22:0) d4(IS) (Unknown) 626.400/264.200 Da - sample 72 of 121 from Ceramides in 6 tissuses First Set 11-5-15
Area: 1.21e+006 counts Height: 5.60e+005 cps RT: 3.77 min
Intensity, cps
Time, min

E

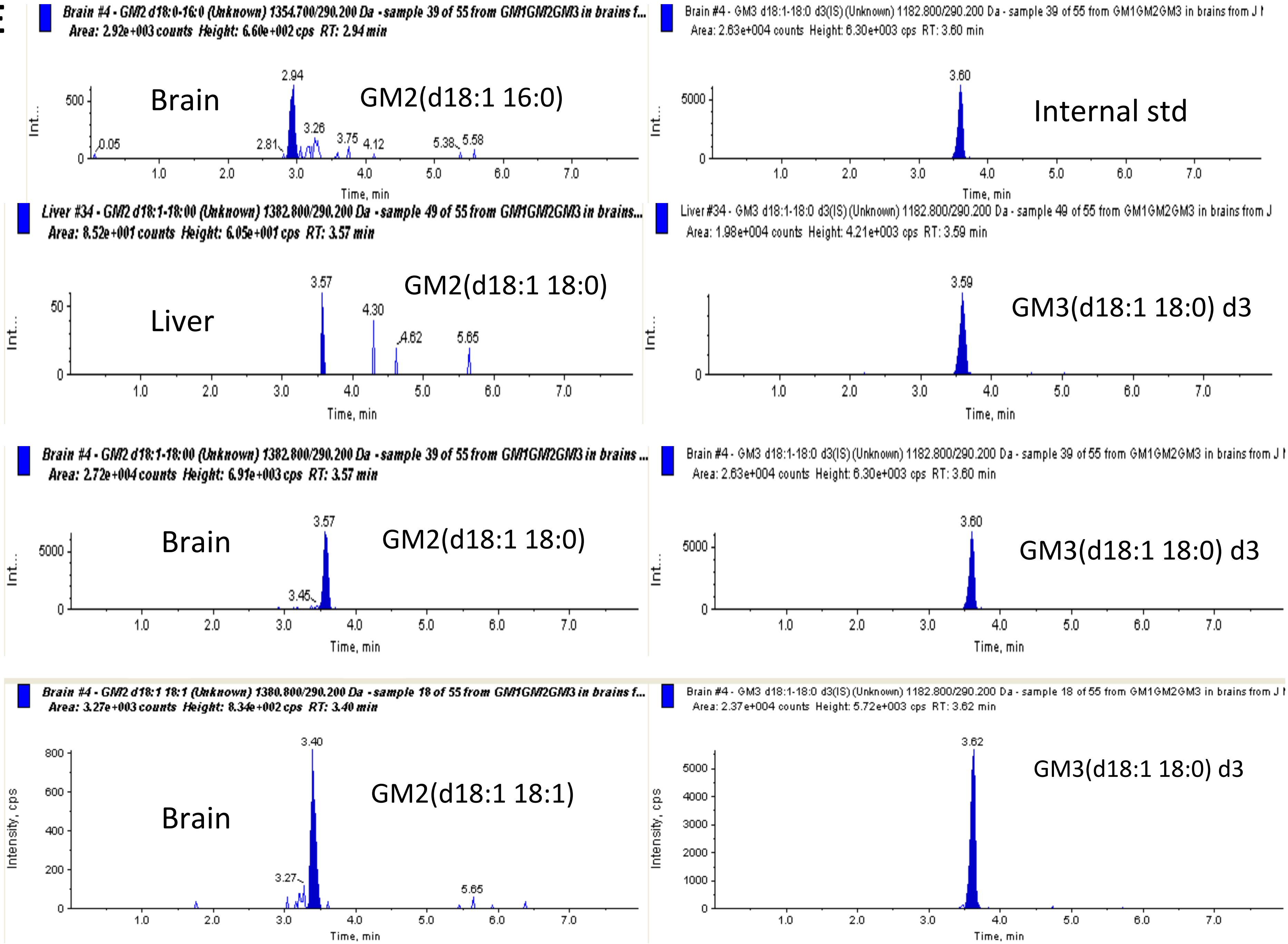

Brain #4 - GM2 d18:0-16:0 (Unknown) 1354.700/290.200 Da - sample 39 of 55 from GM1GM2GM3 in brains f...
Area: 2.92e+003 counts Height: 6.60e+002 cps RT: 2.94 min
Brain
GM2(d18:1 16:0)
Brain #4 - GM3 d18:1-18:0 d3(IS) (Unknown) 1182.800/290.200 Da - sample 39 of 55 from GM1GM2GM3 in brains from J
Area: 2.63e+004 counts Height: 6.30e+003 cps RT: 3.60 min
Internal std
Liver #34 - GM2 d18:1-18:00 (Unknown) 1382.800/290.200 Da - sample 49 of 55 from GM1GM2GM3 in brains...
Area: 8.52e+001 counts Height: 6.05e+001 cps RT: 3.57 min
Liver
GM2(d18:1 18:0)
Liver #34 - GM3 d18:1-18:0 d3(IS) (Unknown) 1182.800/290.200 Da - sample 49 of 55 from GM1GM2GM3 in brains from J
Area: 1.98e+004 counts Height: 4.21e+003 cps RT: 3.59 min
GM3(d18:1 18:0) d3
Brain #4 - GM2 d18:1-18:00 (Unknown) 1382.800/290.200 Da - sample 39 of 55 from GM1GM2GM3 in brains ...
Area: 2.72e+004 counts Height: 6.91e+003 cps RT: 3.57 min
Brain
GM2(d18:1 18:0)
Brain #4 - GM3 d18:1-18:0 d3(IS) (Unknown) 1182.800/290.200 Da - sample 39 of 55 from GM1GM2GM3 in brains from J
Area: 2.63e+004 counts Height: 6.30e+003 cps RT: 3.60 min
GM3(d18:1 18:0) d3
Brain #4 - GM2 d18:1 18:1 (Unknown) 1380.800/290.200 Da - sample 18 of 55 from GM1GM2GM3 in brains f...
Area: 3.27e+003 counts Height: 8.34e+002 cps RT: 3.40 min
Brain
GM2(d18:1 18:1)
Brain #4 - GM3 d18:1-18:0 d3(IS) (Unknown) 1182.800/290.200 Da - sample 18 of 55 from GM1GM2GM3 in brains from J
Area: 2.37e+004 counts Height: 5.72e+003 cps RT: 3.62 min
GM3(d18:1 18:0) d3
Time, min
Intensity, cps
